# Primary Prevention of Gestational Diabetes Mellitus and Large-for-Gestational-Age Newborns by Lifestyle Counseling: A Cluster-Randomized Controlled Trial

**DOI:** 10.1371/journal.pmed.1001036

**Published:** 2011-05-17

**Authors:** Riitta Luoto, Tarja I. Kinnunen, Minna Aittasalo, Päivi Kolu, Jani Raitanen, Katriina Ojala, Kirsi Mansikkamäki, Satu Lamberg, Tommi Vasankari, Tanja Komulainen, Sirkku Tulokas

**Affiliations:** 1 UKK Institute for Health Promotion Research, Tampere, Finland; 2 National Institute for Health and Welfare, Helsinki, Finland; 3 School of Health Sciences, University of Tampere, Finland; 4 Tampere University Central Hospital, Tampere, Finland; Harvard Medical School, United States of America

## Abstract

**Background:**

Our objective was to examine whether gestational diabetes mellitus (GDM) or newborns' high birthweight can be prevented by lifestyle counseling in pregnant women at high risk of GDM.

**Method and Findings:**

We conducted a cluster-randomized trial, the NELLI study, in 14 municipalities in Finland, where 2,271 women were screened by oral glucose tolerance test (OGTT) at 8–12 wk gestation. Euglycemic (*n* = 399) women with at least one GDM risk factor (body mass index [BMI] ≥25 kg/m^2^, glucose intolerance or newborn's macrosomia (≥4,500 g) in any earlier pregnancy, family history of diabetes, age ≥40 y) were included. The intervention included individual intensified counseling on physical activity and diet and weight gain at five antenatal visits. Primary outcomes were incidence of GDM as assessed by OGTT (maternal outcome) and newborns' birthweight adjusted for gestational age (neonatal outcome). Secondary outcomes were maternal weight gain and the need for insulin treatment during pregnancy. Adherence to the intervention was evaluated on the basis of changes in physical activity (weekly metabolic equivalent task (MET) minutes) and diet (intake of total fat, saturated and polyunsaturated fatty acids, saccharose, and fiber). Multilevel analyses took into account cluster, maternity clinic, and nurse level influences in addition to age, education, parity, and prepregnancy BMI. 15.8% (34/216) of women in the intervention group and 12.4% (22/179) in the usual care group developed GDM (absolute effect size 1.36, 95% confidence interval [CI] 0.71–2.62, *p = *0.36). Neonatal birthweight was lower in the intervention than in the usual care group (absolute effect size −133 g, 95% CI −231 to −35, *p = *0.008) as was proportion of large-for-gestational-age (LGA) newborns (26/216, 12.1% versus 34/179, 19.7%, *p = *0.042). Women in the intervention group increased their intake of dietary fiber (adjusted coefficient 1.83, 95% CI 0.30–3.25, *p = *0.023) and polyunsaturated fatty acids (adjusted coefficient 0.37, 95% CI 0.16–0.57, *p<*0.001), decreased their intake of saturated fatty acids (adjusted coefficient −0.63, 95% CI −1.12 to −0.15, *p = *0.01) and intake of saccharose (adjusted coefficient −0.83, 95% CI −1.55 to −0.11, *p  = * 0.023), and had a tendency to a smaller decrease in MET minutes/week for at least moderate intensity activity (adjusted coefficient 91, 95% CI −37 to 219, *p = *0.17) than women in the usual care group. In subgroup analysis, adherent women in the intervention group (*n* = 55/229) had decreased risk of GDM (27.3% versus 33.0%, *p = *0.43) and LGA newborns (7.3% versus 19.5%, *p = *0.03) compared to women in the usual care group.

**Conclusions:**

The intervention was effective in controlling birthweight of the newborns, but failed to have an effect on maternal GDM.

**Trial registration:**

Current Controlled Trials ISRCTN33885819

*Please see later in the article for the Editors' Summary*

## Abstract 2

In a cluster-randomized trial, Riitta Luoto and colleagues find that counseling on diet and activity can reduce the birthweight of babies born to women at risk of developing gestational diabetes mellitus (GDM), but fail to find an effect on GDM.

## Editors' Summary

**Background:**

Gestational diabetes mellitus (GDM) is diabetes that is first diagnosed during pregnancy. Like other types of diabetes, it is characterized by high levels of sugar (glucose) in the blood. Blood-sugar levels are normally controlled by insulin, a hormone that the pancreas releases when blood-sugar levels rise after meals. Hormonal changes during pregnancy and the baby's growth demands increase a pregnant woman's insulin needs and, if her pancreas cannot make enough insulin, GDM develops. Risk factors for GDM, which occurs in 2%–14% of pregnant women, include a high body-mass index (a measure of body fat), excessive weight gain or low physical activity during pregnancy, high dietary intake of polyunsaturated fats, glucose intolerance (an indicator of diabetes) or the birth of a large baby in a previous pregnancy, and a family history of diabetes. GDM is associated with an increased rate of cesarean sections, induced deliveries, birth complications, and large-for-gestational-age (LGA) babies (gestation is the time during which the baby develops within the mother). GDM, which can often be controlled by diet and exercise, usually disappears after pregnancy but increases a woman's subsequent risk of developing diabetes.

**Why Was This Study Done?:**

Although lifestyle changes can be used to control GDM, it is not known whether similar changes can prevent GDM developing (“primary prevention”). In this cluster-randomized controlled trial, the researchers investigate whether individual intensified counseling on physical activity, diet, and weight gain integrated into routine maternity care visits can prevent the development of GDM and the occurrence of LGA babies among newborns. In a cluster-randomized controlled trial, groups of patients rather than individual patients are randomly assigned to receive alternative interventions, and the outcomes in different “clusters” are compared. In this trial, each cluster is a municipality in the Pirkanmaa region of Finland.

**What Did the Researchers Do and Find?:**

The researchers enrolled 399 women, each of whom had a normal blood glucose level at 8–12 weeks gestation but at least one risk factor for GDM. Women in the intervention municipalities received intensified counseling on physical activity at 8–12 weeks' gestation, dietary counseling at 16–18 weeks' gestation, and further physical activity and dietary counseling at each subsequent antenatal visits. Women in the control municipalities received some dietary but little physical activity counseling as part of their usual care. 23.3% and 20.2% of women in the intervention and usual care groups, respectively, developed GDM, a nonstatistically significant difference (that is, a difference that could have occurred by chance). However, the average birthweight and the proportion of LGA babies were both significantly lower in the intervention group than in the usual care group. Food frequency questionnaires completed by the women indicated that, on average, those in the intervention group increased their intake of dietary fiber and polyunsaturated fatty acids and decreased their intake of saturated fatty acids and sucrose as instructed during counseling, The amount of moderate physical activity also tended to decrease less as pregnancy proceeded in the intervention group than in usual care group. Finally, compared to the usual care group, significantly fewer of the 24% of women in the intervention group who actually met dietary and physical activity targets (“adherent” women) developed GDM.

**What Do These Findings Mean?:**

These findings indicate that intensified counseling on diet and physical activity is effective in controlling the birthweight of babies born to women at risk of developing GDM and encourages at least some of them to alter their lifestyle. However, the findings fail to show that the intervention reduces the risk of GDM because of the limited power of the study. The power of a study—the probability that it will achieve a statistically significant result—depends on the study's size and on the likely effect size of the intervention. Before starting this study, the researchers calculated that they would need 420 participants to see a statistically significant difference between the groups if their intervention reduced GDM incidence by 40%. This estimated effect size was probably optimistic and therefore the study lacked power. Nevertheless, the analyses performed among adherent women suggest that lifestyle changes might be a way to prevent GDM and so larger studies should now be undertaken to test this potential primary prevention intervention.

**Additional Information:**

Please access these Web sites via the online version of this summary at http://dx.doi.org/10.1371/journal.pmed.1001036.

- The US National Institute of Diabetes and Digestive and Kidney Diseases provides information for patients on diabetes and on gestational diabetes (in English and Spanish)
- The UK National Health Service Choices website also provides information for patients on diabetes and on gestational diabetes, including links to other useful resources
- The MedlinePlus Encyclopedia has pages on diabetes and on gestational diabetes; MedlinePlus provides links to additional resources on diabetes and on gestational diabetes (in English and Spanish)
- More information on this trial of primary prevention of GDM is available

## Introduction

Gestational diabetes mellitus (GDM) is defined as a type of diabetes first diagnosed during pregnancy [1]. Incidence of GDM varies from 2% to 14% globally and it is increasing [2]. Maternal glucose has been associated with a risk of adverse pregnancy outcomes in a linear manner [3]. Borderline GDM has been linked with higher rates of cesarean sections and induced deliveries, shoulder dystocia and birth injuries [4], and pathogenesis in the offspring of overweight and metabolic syndrome [5].

High intake of saturated fat, low intake of polyunsaturated fat, and excessive gestational weight gain may increase the risk of GDM [6]–[10]. Physical activity is also associated with decreased risk of GDM [11]. Lifestyle modifications have been shown to be a valuable adjunctive therapy of GDM [12] but to date there are no adequately powered trials on primary prevention of GDM. Earlier studies on prevention of gestational weight gain by dietary and physical activity counseling have found favorable results [13] and structured aerobic exercise training has been shown to decrease birthweight of the newborns [14]. In our own pilot study, intensive lifestyle counseling produced favorable changes both in diet and physical activity [15],[16]. The aim of this cluster-randomized trial was to examine whether individual intensified counseling on physical activity, diet, and weight gain integrated into routine maternity care visits could prevent the development of GDM and newborns' high birthweight adjusted for gestational age.

## Methods

### Design and Study Population

The methods of this study have been described in detail previously (Text S1 and S2) [17]. The study is a cluster-randomized trial conducted in maternity clinics of primary health care centers of 14 municipalities in Pirkanmaa region in south-western Finland. All 14 municipalities with at least 70 annual deliveries were recruited to the study. The unit of randomization was municipality. In the randomization process, participating municipalities were first pairwise matched with regard to annual number of births, size and socio-economic level of the population, estimated incidence of GDM, and urbanity level. Municipalities were then randomized by computer. The rationale for using cluster randomization was to avoid contamination between trial arms, which would have occurred if individuals or clinics were randomized. All 53 nurses working in maternity clinics in these municipalities recruited pregnant women at 8–12 wk gestation between 1 October 2007 and 31 December 2008. The study was completed at the end of 2009, when all participating women had given birth.

Pregnant women were eligible to enter the study if they had at least one of the following risk factors: body mass index (BMI) ≥25 kg/m^2^ based on measured height and self-reported prepregnancy weight; GDM or any signs of glucose intolerance or newborn's macrosomia (≥4,500 g) in any earlier pregnancy; type 1 or 2 diabetes in first- or second-degree relatives; or age ≥40 y. Women were excluded if they had any of the following: at least one of the three baseline (8–12 wk gestation) oral glucose tolerance test (OGTT) measurements was abnormal (fasting blood glucose ≥5.3 mmol/l, >10.0 mmol/l at 1 h, and >8.6 mmol/l at 2 h) [1]; prepregnant type 1 or 2 diabetes; inability to speak Finnish; age <18 y; multiple pregnancy; physical restriction preventing physical activity; substance abuse; treatment or clinical history for psychiatric illness.

The research protocol was approved by the Urho Kekkosen Kuntoinstituuttisäätiö institute review board, the ethical committee of Pirkanmaa Hospital District, and the physicians in charge of primary health care in the 14 municipalities. All participants provided written informed consent.

### Intervention

The intervention continued from the first maternity clinic visit at 8–12 wk gestation until 37 wk gestation (Text S1) [17]. At the first visit the recommendations for gestational weight gain [18] were discussed and an appropriate weight gain graph was selected from the follow-up notebook to guide the participant in monitoring her weight gain. The primary physical activity counseling was implemented at 8–12 wk gestation and the primary dietary counseling session at 16–18 wk gestation. The rationale for implementing physical activity and dietary counseling at separate visits was 2-fold: the nurses' time for counseling was limited to 2 h in total for the first visit and thus only one primary counseling focus could be included. Secondly, the nurses were more familiar with dietary issues and, therefore, including physical activity counseling in the first visit emphasized the difference between usual care. Physical activity counseling was enhanced at four, and diet counseling at three subsequent visits. If OGTT was pathological at 26–28 wk gestation, women were additionally referred to other health care specialists.

Aims of the physical activity counseling were to increase leisure time physical activity of those pregnant women who were not fulfilling the physical activity recommendations to the recommended level for health [19] and to maintain or adjust leisure time physical activity of those women who were already fulfilling the recommendations. The minimum weekly leisure time physical activity dose, including also light-intensity physical activity, entered progressively in the plan was 800 MET (multiples of resting metabolic equivalents) minutes. This amount is in line with Haskell et al. [20], suggesting after the initiation of this study the maximum of 750 MET minutes of moderate-intensity physical activity for health. During the primary visit the participants were offered an opportunity to participate in monthly thematic meetings on physical activity including group exercise.

Evaluation of leisure-time physical activity was based on a validated self-report [21].

Based on Finnish dietary recommendations, the goal of dietary counseling was to help participants achieve a healthy diet containing ≤10% saturated fat, 5%–10% polyunsaturated fat, 25%–30% total fat, and <10% saccharose of total energy intake, and 25–35 g/d fiber [22]. A study that was successful in preventing type 2 diabetes mellitus in Finland was used as a reference [23]. In practice, the participants were advised (1) to consume vegetables, fruits, and berries, preferably at least five portions (400 g) a day; (2) to select mostly high fiber bread (>6 g fiber/100 g) and other whole-meal products; (3) to select mostly fat-free or low-fat versions of milk and milk products (e.g., yoghurt, cheese, ice cream) and of meat and meat products; (4) to eat fish at least twice per week (excluding the fish species not recommended for pregnant women); (5) to use moderate amounts of soft table spreads on bread, oil-based salad dressing in salad, and oil in cooking and baking; (6) to consume seldom and only in small-portions foods high in fat; and (7) to consume seldom and only in small-portions snacks containing high levels of sugar and/or fat (e.g., sweets, high-sugar drinks, cookies, ice cream, sweet and savoury pastries, potato chips) [24].

Counseling cards helped the nurses to standardize their counseling. The participants used follow-up notebooks to set their individual plans for physical activity and dietary changes and to a keep record of their adherence to their plans.

Women in the usual care group received no counseling beyond usual care, which includes some dietary counseling (partly on different topics) and follow-up of gestational weight [15], but only little physical activity counseling [16]. All nurses were supported with meetings every third month during the intervention.

### Outcome Variables and Data Collection

The two primary outcomes of the trial were the proportion of women with GDM based on 26–28 gestation week OGTT (maternal outcome) and the newborns' birthweight adjusted for gestational age (neonatal outcome). Diagnosis of GDM was based on the OGTT (at least one of the following criteria was met: fasting blood glucose ≥5.3 mmol/l, >10.0 mmol/l at 1 h, and >8.6 mmol/l at 2 h) [1].

In addition to this definition, we also combined the birthweight of the newborn (≥4,500 g or >4,000 g) or possible use of insulin or other diabetic medication during pregnancy to two variations of GDM diagnosis, since the OGTT might have been pathological after 26–28 wk gestation. The rationale for using birthweight was that performing third OGTT measurement at 37–39 wk gestation was not feasible. The information on the outcomes was based both on hospital records and measurements made by the research group.

Level of glucose intolerance and insulin resistance was based on the homeostasis model assessment insulin resistance (HOMA-IR) calculator [25] and calculated as fasting insulin concentration (µU/ml) × fasting glucose concentration (mmol/l)/22.5. Blood samples for determination of glucose intolerance and insulin resistance were taken at 8–12 and 26–28 wk gestation.

Neonatal outcomes to be reported in this article are sex of the newborn, proportions of macrosomic (≥4,500 g) and large-for-gestational-age (LGA) and small-for-gestational-age (SGA) newborns, gestational age at delivery, birthweight standard deviation (SD) score, crown-heel length and crown-heel length SD score, ponderal index, and newborn head circumference. LGA refers to an infant whose birthweight is above the 90th percentile adjusted gestational age and SGA for an infant whose birthweight is below the 10th percentile adjusted gestational age. Birthweight percentiles and SD scores were based on Medical Birth Registry information on Finnish children born during years 2004–2006 and the method used by Kramer et al. [26]. Crown-heel length is the distance from the crown of the head to the heel and measured with a measuring board. Head circumference is measured with measuring tape and obtained to the nearest millimeter. Ponderal index was calculated as birthweight in kilograms divided by the cube of the crown-heel length in meters. Measurements for all neonatal outcomes were performed immediately after birth by the hospital midwifes and the information was gathered for the study from maternal records.

Other secondary outcomes were (1) gestational weight gain calculated on the basis of self-reported prepregnancy weight and the last measured weight during pregnancy in the maternal care, (2) the need for insulin or other diabetic medication from 26–28 wk gestation onwards, and (3) child weight development after delivery, which will be reported in a separate article. In this article we also report the proportion of women with pre-eclampsia determined as protein in the urine sample assessed by urine testing stick after 20 wk gestation and systolic blood pressure ≥140 mmHg and diastolic blood pressure ≥90 mmHg. All this information was obtained from maternal records. Additionally, data on physical activity and diet was collected by validated questionnaires at 8–12 (questions covering the 1-mo period before pregnancy), 26–28, and 36–37 wk gestation (Text S1) [17],[21],[24].

Evaluation of leisure-time physical activity was based on a validated self-report (21) at baseline, 26–28 wk gestation, and 36–37 wk gestation. At baseline, physical activity prior to pregnancy was inquired. At follow-ups, the questions concerned physical activity during the past 3 wk. The degree of breathlessness (strong, some, none) was used to help the women to determine the intensity of their physical activity. In quantifying MET minutes, 3 METs were used for light, 5 METs for moderate, and 7 METs for vigorous physical activity. In the analysis, moderate and vigorous MET minutes were summed to form MET minutes for at least moderate physical activity. Dietary habits were assessed by using a validated 181-item food frequency questionnaire [24] at baseline, 26–28 wk gestation, and 36–37 wk gestation. At baseline, the women were asked questions about their diet during 1 mo prior to the pregnancy, since their diet may have changed due to nausea or vomiting at the beginning of the pregnancy. In the follow-up, the women were asked questions about their diet during the previous month.

Behavior changes, expected to contribute to the possible effects of the intervention on the outcomes, were changes in leisure-time physical activity (weekly MET minutes) and diet (intake of total fat, saturated and polyunsaturated fatty acids, saccharose, and fiber).

Women were defined to be adherent to the recommendations, if they fulfilled at least four of the five dietary aims and/or their physical activity exceeded 800 MET minutes/wk at 36–37 wk gestation and their total weight gain did not exceed their BMI-specific limits.

The nurses elicited information on selected adverse effects [27] by a structured interview at four of the visits. Adverse effects included nausea, bleeding, painful contractions, dizziness, breathlessness, headache, chest pain, tiredness/fatigue, calf pain, or musculoskeletal problems. The response alternatives were “no,” “sometimes,” and “often.”

### Sample Size

The power calculations for this study were based on the assumption of detecting a 40% reduction in the incidence of GDM (GDM incidence 40% in the usual care municipalities and 24% in the intervention municipalities). With these incidences of GDM, the power of the study would be 0.80, significance level 0.05, and coefficient of variation of rate between clusters, indicating cluster sampling, 0.1. On the basis of these assumptions, 420 women were needed for the analyses. As we expected a drop-out rate similar to the one in our pilot study (25%), we needed to recruit a total of 560 women to the study.

### Statistical Analysis

Statistical analysis was performed by intention to treat approach in the originally assigned groups. Numbers and percentages are reported for categorical variables and means with standard deviations (SDs) or 95% confidence intervals (CIs) for continuous variables. Statistical analyses for adverse effects were performed using SPSS software (version 17.0.1) and adverse effect multilevel analyses by using STATA (version 11.0). The main method for between-group differences was multilevel analysis enabling simultaneous examination of cluster, clinic, nurse, and individual-level influences on outcomes and the correction of results for between-cluster, between-clinic, and between-nurse variation.

The effects of the intervention on maternal and neonatal outcomes and changes in physical activity and diet were analyzed by using STATA's generalized linear latent and mixed models (GLLAMM) and multilevel mixed-effects linear regression (xtmixed) by fitting four-level random effects models. Individual-level variables included as covariates in the multilevel models were age (continuous), education (low, basic or secondary education; medium, polytechnic; high, university degree), sex of the infant, parity (0 or ≥1), prepregnancy BMI (continuous), smoking (never, before pregnancy only, before and during pregnancy). Total gestational weight gain was not included in the multilevel model since it belongs to the causal pathway of the effect of lifestyle counseling. Gestational age in days was included in analyzing the abnormal OGTT at 26–28 wk gestation, because a higher proportion of women in the usual care group (21.4%) than in the intervention group (16.5%) underwent OGTT before 26 wk gestation. Four outliers were excluded from analyses: women with BMI 48.5 or 40.4 (two women in the intervention group) and women whose newborns birthweight were 740 g (one woman in the intervention group) or 850 g (one woman in the usual care group). However, including outliers to the analyses did not change the results. The incidence of GDM and LGA was also calculated by including only adherent women in the intervention group to the analyses and estimating the association by using chi-square statistics.

## Results

### Baseline

**Figure 1:**
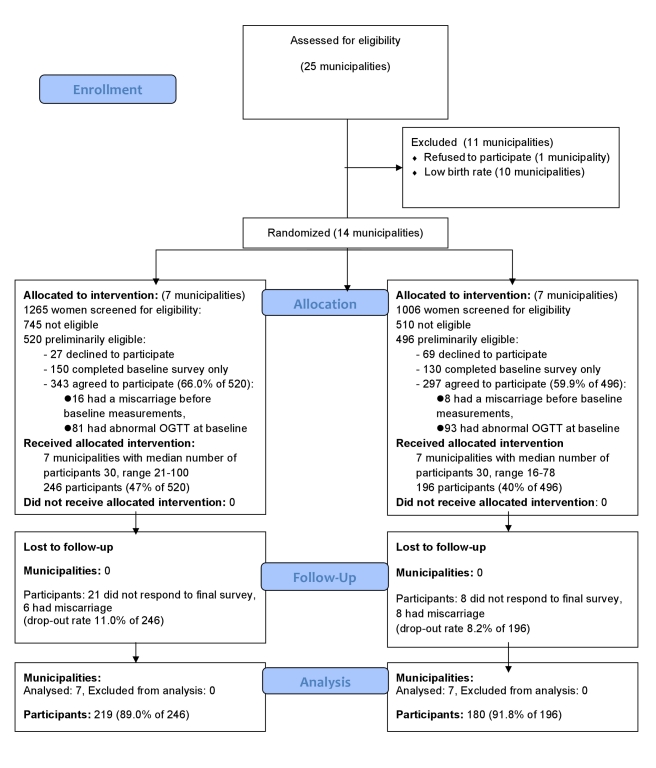
CONSORT flow diagram of the cluster-randomized trial.

Of the 2,271 women screened, 520 (22.9%) in the intervention group and 496 (21.8%) in the control group were preliminarily eligible for the study. Of them, 343 (66.0%) in the intervention and 297 (59.9%) in the usual care group agreed to participate in the trial (Figure 1). However, 81 (23.6%) of the participants in intervention group and 93 (31.3%) of the participants in the usual care group had an abnormal OGTT result at baseline (8 to 12 wk gestation) and were thus excluded. The final number of participants in the analyses was 219 (89.0% of participants receiving allocated intervention) in the intervention group and 180 (91.8% of participants receiving allocated intervention) in the usual care group. The intervention and the usual care group had an equal proportion of women fulfilling one (68.5% versus 66.5%), two (28.3% versus 27.9%), or three of the inclusion criteria (2.7% versus 4.5%).

The mean age of the women was 30 y and 47% of them were primiparous in the intervention and 41% in the usual care group (Tables 1 and S1). Average BMI before pregnancy was 26 kg/m^2^ in both groups. There were more women in the intervention group (26.8%) with high education than in the usual care group (20.6%). Being overweight or having a family history of diabetes were the most common reasons for inclusion (Table S1).

**Table 1:** Baseline characteristics of the study population.

Characteristics	Intervention Group (*n* = 219)^a^	Usual Care Group (*n* = 180)^a^
Age (y)	29.5±4.8	30.0±4.7
*n* Primiparous (%)	103 (47.0)	73 (40.6)
BMI before pregnancy	26.3±4.9	26.4±4.3
Range of BMI before pregnancy^b^	17.0–48.5	17.2–37.8
*n* Education (%)^c^		
High	58 (26.8)	36 (20.6)
Medium	85 (39.4)	80 (45.7)
Low	73 (33.8)	59 (33.7)
*n* Smoking before or during pregnancy (%)^d^	44 (20.9)	45 (26.2)
Inclusion criteria (%):		
Overweight (BMI >25 kg/m^2^)	128 (58.4)	110 (61.5)
Macrosomia in previous pregnancies	6 (2.7)	5 (2.8)
Earlier gestational glucose intolerance/GDM	26 (11.9)	19 (10.6)
Age ≥40 y	5 (2.3)	5 (2.8)
Family history of diabetes	126 (57.5)	90 (50.3)

a Plus/minus values are means ± SD.

b Outliers of 48.5 and 40.4 in BMI excluded from further analyses.

c High, university degree; medium, polytechnic education; low, basic or secondary education.

d Information on smoking missing *n* = 16.

### Primary Outcomes

There were no significant differences between the intervention and the usual care group at baseline or at 26–28 wk gestation in glucose intolerance measurements (Table 2). The proportion of women with GDM based on different criteria did not differ between the groups (Table 3). Total gestational weight gain, pre-eclampsia, or use of diabetic medication did not differ significantly between the groups (Table 3).

**Table 2:** Means and SD for variables related to the OGTT, insulin, and homeostatic model insulin resistance at 8–12 and 26–28 wk gestation.

Variables	8–12 wk Gestation			26–28 wk Gestation			Change from 8–12 to 26–28 wk Gestation		
	Mean±SD		*p*-Value	Mean±SD		*p*-Value	Mean±SD		*p*-Value
	Intervention Group (*n = *219)	Usual Care Group (*n = *180)	Difference between Groups^a^	Intervention Group (*n = *219)	Usual Care Group (*n = *180)	Difference between Groups^a^	Intervention Group (*n = *219)	Usual Care Group (*n = *180)	Difference between Groups^a^
Glucose levels in 2-h OGTT (mg/l)									
Fasting (0)	4.90±0.22	4.89±0.26	0.68	4.74±0.33	4.77±0.32	0.44	—	—	—
1 h	6.43±1.55	6.18±1.37	0.09	7.70±1.74	7.47±1.77	0.23	—	—	—
2 h	5.41±1.07	5.25±0.94	0.12	5.92±1.21	5.87±1.19	0.99	—	—	—
Insulin	11.55±5.92	11.22±5.69	0.70	13.37±6.48	12.31±6.25	0.10	1.79±6.05	0.97±5.70	0.23
HOMA-IR^b^	1.47±0.72	1.46±0.69	0.86	1.69±0.80	1.57±0.77	0.13	0.21±0.75	0.11±0.70	0.24

a Mixed-effects linear regression models for variables in each row separately, adjusted for gestational age (8–12 or 26–28 wk gestation). Multilevel analysis.

b Outliers 62.5 (intervention) and 63.9 (usual care) in insulin were excluded.

HOMA-IR, homeostatic model insulin resistance.

**Table 3:** Maternal and neonatal outcomes of the trial.

Outcomes	Intervention Group (*n = *216)	Usual Care Group (*n = *179)	*n* Missing	Absolute Effect Size^a^ (95% CI)	*p*-Value^b^	Adjusted^c^ Effect Size (95% CI)	Adjusted^c^ *p*-Value
*Maternal*							
*n* GDM I (%)^d^	50 (23.3)	36 (20.2)	2	1.22 (0.69–2.15)	0.49	1.18 (0.67–2.07)	0.57
*n* GDM II (%)^e^	73 (34.0)	59 (33.1)	2	1.06 (0.67–1.68)	0.81	1.04 (0.65–1.65)	0.87
*n* GDMIII (%)^f^	44 (20.5)	29 (16.3)	2	1.34 (0.76–2.39)	0.31	1.27 (0.72–2.24)	0.40
*n* At least one abnormal value in 2 h OGTT test at 26–28 wk gestation	34 (15.8)	22 (12.4)	2	1.36 (0.71–2.62)	0.36	1.61 (0.83–3.14)	0.16
Gestational weight gain^a^ (kg)	13.8±5.8	14.2±5.1	31	−0.39 (−1.58 to 0.80)	0.52	−0.43 (−1.52 to 0.67)	0.44
*n* Insulin or other diabetic medication (%)	7 (3.7)	8 (5.1)	49	0.72 (0.25–2.02)	0.53	0.64 (0.22–1.88)	0.41
*n* Pre-eclampsia (%)	14 (6.5)	10 (5.9)	11	1.12 (0.48–2.59)	0.79	1.32 (0.53–3.31)	0.55
*Neonatal*							
Birthweight (g)	3,532±514	3,659±455	7	−133 (−231 to −35)	0.008	−104 (−201 to −7.6)	0.035
Birthweight/gestational age (g/wk gestation)	89.5±11.2	92.4±10.8	7	−3.08 (−5.29 to −0.87)	0.006	−2.54 (−4.75 to −0.33)	0.024
Birthweight SD score	0.18±0.94	0.32±0.98	7	−0.16 (−0.35 to 0.04)	0.11	−0.15 (−0.35 to 0.04)	0.12
*n* Large for gestational age (%)	26 (12.1)	34 (19.7)	7	0.56 (0.32–0.98)	0.042	0.55 (0.30–0.98)	0.043
*n* Macrosomia (birthweight >4,500 g) (%)	7 (3.3)	8 (4.6)	7	0.69 (0.20–2.42)	0.57	0.65 (0.13–3.38)	0.61
*n* Macrosomia (birthweight >4,000 g) (%)	37 (17.2)	36 (20.8)	7	0.79 (0.47–1.34)	0.38	0.83 (0.47–1.47)	0.53
*n* Small for gestational age (%)	10 (4.7)	5 (2.9)	7	1.64 (0.55–4.89)	0.38	1.89 (0.56–6.37)	0.31
Crown-heel length (cm)	50.4±2.1	50.7±2.0	9	−0.37 (−0.79 to 0.04)	0.078	−0.32 (−0.74 to 0.10)	0.13
Crown-heel length SD score	0.27±0.92	0.36±0.98	9	−0.09 (−0.28 to 0.10)	0.36	−0.11 (−0.31 to 0.08)	0.25
Ponderal index (weight, kg/height, m^3^)	27.6±2.5	28.0±2.2	9	−0.37 (−0.92 to 0.17)	0.18	−0.25 (−0.74 to 0.24)	0.32
Head circumference (cm)	35.3±1.4	35.5±1.8	14	−0.22 (−0.54 to 0.10)	0.19	−0.14 (−0.46 to 0.18)	0.41

Continuous dependent variables from multilevel mixed-effects linear regression models (results as means and 95% CIs) and categorized variables from multilevel logistic regression models (results as odds ratios and 95% CIs).

a Plus-minus values are means ± SD. Four outliers excluded from analyses: BMIs of 48.5 kg/m ^2^ and 40.4 kg/m ^2^, birthweights of 740 g and 850 g.

b Unadjusted analysis.

c Multilevel analysis adjusted for age (continuous), education (high/medium/low),sex of the infant, parity (primiparous/multiparous), prepregnancy BMI (continuous), and smoking (yes, during or before pregnancy, and/or not during or before pregnancy).

d Criteria for GDM: at least one abnormal value in 2 h OGTT or newborn birthweight equal or higher than 4,500 g or use of insulin or other diabetic medication.

e Criteria for GDM: at least one abnormal value in 2 h OGTT or newborn birthweight equal or higher than 4,000 g or use of insulin or other diabetic medication.

f Criteria for GDM: at least one abnormal value in 2 h OGTT or use of insulin or other diabetic medication.

Among the newborns, the proportion of males was 54.0% in the intervention and 43.1% in the usual care group. Gestational age at delivery was similar in both groups (39.4±1.9 wk versus 39.6±1.3 wk). The average newborns' birthweight was lower in the intervention group than in the usual care group (3,532 g versus 3,659 g, adjusted *p* = 0.035) (Table 3). Between-group differences in birthweight (absolute effect size −133 g, 95% CI −231 to −35) and in birthweight per gestational age (absolute effect size −3.08, 95% CI −5.29 to −0.87) remained significant after taking cluster, clinic, nurse, maternal age, education, sex of the infant, parity, prepregnancy BMI, gestational weight gain, and smoking into account (*p* = 0.024). Birthweight SD score was not significantly different between groups. The intracluster correlation coefficient (ICC) for birthweight was 0.02, when adjusted for all covariates. Newborns' birthweight did not differ significantly between women with one or more inclusion criteria.

The proportion of LGA infants was lower in the intervention (12.1%) than in the usual care group (19.7%, *p = *0.042) and the statistical significance persisted after adjusting for individual-level covariates (*p = *0.043) (Table 3). There were no statistically significant differences between the groups in the proportion of macrosomic infants, crown-heel length, crown-heel length SD score, SGA, ponderal index, or head circumference. Since there were only four infants with low birthweight (<2,500 g), those results are not shown.

### Behavior Change

Women in the intervention group had a tendency to a smaller decrease in at least moderate activity MET minutes (adjusted coefficient 91, 95% CI −37 to 219, *p = *0.17) than women in the usual care group from baseline to 26–28 wk gestation (Table 4). The intervention group reduced their intake of saturated fatty acids from baseline to 26–28 wk gestation as compared to the usual care group (adjusted coefficient −0.56, 95% CI −0.95 to −0.17, *p = *0.005) and a similar, but statistically nonsignificant change was observed in total fat intake (Table 4). Intervention group also differed from usual care group in decreased use of saccharose (adjusted coefficient −0.59, 95% CI −1.16 to −0.03, *p = *0.04) at 26–28 wk gestation. When comparing the between-group differences in changes from baseline to 36–37 wk gestation, the intervention group had reduced their intake of saturated fatty acids (coefficient −0.63, 95% CI −1.12 to −0.15, *p = *0.01) and saccharose (adjusted coefficient −0.83, 95% CI −1.55 to −0.11, *p = *0.023) and increased their intake of dietary fiber (coefficient 1.83, 95% CI 0.30–3.35, *p = *0.019) and polyunsaturated fatty acids (coefficient 0.37, 95% CI 0.16–0.57, *p<*0.001).

**Table 4:** Physical activity and dietary changes from baseline to 26–28 and 36–37 wk gestation in the intervention and the usual care groups.

Physical Activity and Dietary Changes	Baseline Before Pregnancy		26–28 wk Gestation		Group Difference in Change from Baseline to 26–28 wk Gestation^a^		36–37 wk Gestation		Group Difference in Change from Baseline to 36–37 wk Gestation^a^	
	Intervention (*n = *212–216)	Usual Care (*n = *166–177)	Intervention (*n = *208–211)	Usual Care (*n = *177–178)	Adjusted Coefficient/OR (95% CI)	Adjusted *p*-Value	Intervention (*n = *179–190)	Usual Care (*n = *155–158)	Adjusted Coefficient/OR (95% CI)	Adjusted *p*-Value
Physical activity										
Total MET min/wk, mean (SD)	1,453 (1,253)	1,785 (1,273)	1,160 (823)	1,233 (1,154)	84 (−97 to 265)	0.36	963 (837)	1,149 (1,127)	−56 (−280 to 168)	0.63
MET min/wk for at least moderate activity, mean (SD)	962 (1,004)	1,193 (1,037)	714 (623.7)	703 (789)	91 (−37 to 219)	0.17	496 (552)	519 (756)	15 (−120 to 151)	0.82
MET min/wk for light activity, mean (SD)	486 (659)	617 (758)	447 (513)	533 (641)	−31 (−141 to 78)	0.57	470 (568)	621 (603)	−80 (−193 to 34)	0.17
≥800 MET min/wk, %	71.2	83.1	62.9	60.4	1.31 (0.82 to 2.09)	0.27	47.3	56.4	0.85 (0.53 to 1.37)	0.51
Diet										
Total energy intake (MJ/d)	9.5 (2.5)	10.3 (3.0)	9.5 (2.4)	9.9 (2.7)	0.01 (−0.42 to 0.43)	0.97	9.3 (2.4)	9.8 (3.1)	−0.03 (−0.51 to 0.45)	0.90
Total energy intake (kcal/d)	2,274 (592)	2,467 (727)	2,278 (581)	2,366 (652)	1.7 (−99 to 103)	0.97	2,221 (573)	2,347 (749)	−7.6 (−122 to 107)	0.90
Protein (E%)^b^	18.3 (2.2)	18.1 (2.4)	18.2 (2.0)	17.8 (2.4)	0.39 (−0.07 to 0.85)	0.094	18.2 (2.2)	17.9 (2.5)	0.23 (−0.19 to 0.65)	0.29
Carbohydrates (E%)	47.4 (5.0)	47.0 (5.7)	48.5 (4.9)	47.9 (4.7)	0.14 (−0.77 to 1.06)	0.76	47.9 (5.4)	47.8 (5.6)	−0.28 (−1.33 to 0.77)	0.60
Saccharose (E%)	9.7 (3.1)	10.4 (3.8)	10.4 (3.3)	11.0 (3.1)	−0.59 (−1.16 to −0.03)	0.04	10.2 (3.6)	11.1 (4.1)	−0.83 (−1.55 to −0.11)	0.023
Dietary fiber (g/d)	24.6 (8.6)	25.4 (8.5)	25.5 (9.0)	25.3 (8.9)	0.56 (−0.86 to 1.97)	0.44	25.0 (8.6)	23.5 (8.3)	1.83 (0.30 to 3.35)	0.019
Total fat (E%)	32.3 (4.4)	32.9 (4.9)	32.2 (4.4)	33.2 (4.0)	−0.55 (−1.29 to 0.20)	0.15	32.8 (4.8)	33.2 (4.9)	−0.08 (−0.99 to 0.82)	0.86
Saturated fatty acids (E%)	12.5 (2.6)	13.1 (3.1)	12.3 (2.4)	13.3 (2.7)	−0.56 (−0.95 to −0.17)	0.005	12.5 (2.6)	13.5 (3.2)	−0.63 (−1.12 to −0.15)	0.01
Trans fatty acids (E%)	0.36 (0.09)	0.37 (0.10)	0.35 (0.09)	0.36 (0.10)	−0.00 (−0.02 to 0.01)	0.65	0.35 (0.09)	0.37 (0.11)	−0.01 (−0.03 to 0.01)	0.30
Monounsaturated fatty acids (E%)	11.9 (1.9)	11.9 (1.8)	12.0 (1.9)	12.1 (1.6)	−0.00 (−0.33 to 0.33)	0.99	12.2 (2.2)	12.1 (2.0)	0.14 (−0.27 to 0.55)	0.51
Polyunsaturated fatty acids (E%)	5.0 (1.0)	5.0 (1.0)	5.1 (0.9)	4.9 (0.9)	0.11 (−0.06 to 0.29)	0.21	5.2 (1.1)	4.8 (1.0)	0.37 (0.16 to 0.57)	<0.001

Continuous dependent variables from multilevel mixed-effects models. Multilevel mixed-effects linear regression (xtmixed) for continuous variables and multilevel mixed-effects logistic regression (GLLAMM) for dichotomous variables. Group differences have been adjusted for baseline value of corresponding dependent variable.

a Group differences have been adjusted for baseline value of corresponding dependent variable, age, education, parity (nulliparous/other), and prepregnancy BMI.

b E%, percentage of total energy intake.

### Incidence of the Primary Outcomes in Adherent Women

The adherent women in the intervention group had less GDM (27.3% [15/55] versus 33.0% [59/179], *p = *0.43) and lower proportion of LGA newborns (7.3% [4/55] versus 19.5 [34/174], *p = *0.03) when compared to all women in the usual care group (not shown in the table).

### Adverse Effects

The intervention group did not statistically significantly differ in any of the selected adverse effects when maternal age and all covariates were taken into account, except in headache at 32–34 wk gestation (41.5% among intervention group versus 56.5% among usual care group, adjusted *p = *0.019) (Table 5).

**Table 5:** Incidence of selected adverse effects (%) among intervention and usual care groups by gestation week.

Adverse Effects	Gestation week											
	16–18			22–24			32–34			37–39		
	Intervention group (*n = *208)	Usual care group (*n = *118)	*p*-Value	Intervention group (*n = *206–207)	Usual care group (*n = *114–116)	*p*-Value	Intervention group (*n = *202–205)	Usual care group (*n = *113–116)	*p*-Value	Intervention group (*n = *201–202)	Usual care group (*n = *117–118)	*p*-Value
Nausea	42.8	42.4	0.38	42.5	42.6	0.45	15.6	18.3	0.53	18.8	13.6	0.23
Bleeding	6.7	3.4	0.15	6.8	3.4	0.15	2.4	0.9	0.26	3.5	3.4	0.77
Painful contractions	7.7	5.1	0.43	7.7	5.2	0.45	12.7	12.2	0.61	29.2	23.7	0.12
Dizziness	38.0	38.1	0.95	38.2	39.1	0.82	23.0	30.4	0.15	16.8	19.5	0.58
Breathlessness	17.3	18.6	0.64	17.5	19.0	0.66	16.6	17.2	0.53	20.8	22.9	0.64
Headache	61.5	61.9	0.87	61.8	62.1	0.85	41.5	56.5	0.019	26.2	32.2	0.19
Chest pain	3.9	3.4	0.87	3.9	3.4	0.86	2.4	2.6	0.89	2.0	0.0	—
Tiredness/fatigue	42.8	49.2	0.90	42.2	48.7	0.86	26.8	33.9	0.70	31.7	34.8	0.97
Calf pain	4.8	9.3	0.20	4.8	8.0	0.48	7.8	12.1	0.29	8.9	10.2	0.88
Musculoskeletal pain	29.3	29.7	0.58	29.6	28.9	0.48	34.2	37.4	0.26	35.3	33.3	0.61

The proportions include women who reported the symptoms sometimes or often. Multilevel analysis adjusted for age, education, BMI at baseline, parity, weight gain, and smoking before or during pregnancy.

## Discussion

Our study evaluated the effectiveness of lifestyle counseling in primary prevention of GDM among a group of euglycemic women with at least one risk factor of GDM. Using a cluster-randomized controlled design, lifestyle counseling was effective in controlling the proportion of LGA newborns, but the result concerning GDM was inconclusive. The intervention had beneficial effects on four of the five dietary aims, i.e., the intake of dietary fiber, saccharose, and saturated and polyunsaturated fatty acids. Additionally, a statistically nonsignificant tendency for lower decrease in at least moderate activity MET minutes by 26–28 wk gestation was observed among the intervention group as compared to the usual care group. Women adherent to the lifestyle aims had lower proportion of LGA newborns and a tendency to lower incidence of GDM.

Earlier studies related to GDM have studied, for example, thresholds for treatment [21],[22] or prevention of pregnancy-related weight gain, and reported some results related to GDM [13],. Landon and colleagues' [29] trial included women with mild GDM, and an Australian trial [30] women with definite GDM at 24–34 wk gestation. Both trials had composite outcomes including perinatal mortality and neonatal complications associated with maternal hyperglycemia [29],[30]. In our study the main outcomes were GDM and LGA newborns, both reflecting maternal hyperglycemia. Studies in which newborn birthweight has been a primary outcome have shown that treatment of maternal gestational hyperglycemia can be beneficial on newborns' birthweight [28]. In an observational hyperglycemia and adverse pregnancy outcomes (HAPO) study [3], maternal blood glucose levels were associated with LGA (birthweight above 90th percentile). In our study, no significant differences between the groups in neonatal ponderal index, total gestational weight gain, macrosomia, or head circumference was discovered. This finding may also be due to the fact that power calculations were based on the main outcome—incidence of GDM. The calculations were not based on earlier trials, since there were no similar studies available at the time of the initiation of the study. The proportion of women with GDM among the intervention group was expected to be half lower than in the usual care group according to the power calculations, but the results did not support reduction. Although the target sample size (*n* = 420) was almost met, the expected difference of 40% in the incidence of GDM between the groups was most likely too ambitious and thus the study lacked sufficient power on the GDM outcome. The proportion of excluded women with abnormal OGTT already at the 8–12 wk gestation was unexpectedly high (23.6% in the intervention group, 31.3% in the usual care group), which decreased our sample size. The recruitment process was 6 mo longer than initially planned, since pregnant women were reluctant to participate in the lifestyle modification program because of lack of time or other personal reasons.

GDM treatment trials have used different intervention strategies: use of insulin, self-monitoring of glucose, and dietary intervention [28]. Lifestyle counseling, in terms of physical activity, diet, and weight gain, has not been incorporated as an intervention strategy in GDM prevention or treatment in any adequately powered study. Physical activity is, however, known to have acute effects on blood glucose and insulin sensitivity during a GDM pregnancy [30], which may further lead to favorable effects on newborns' birthweight. Small studies with instructed exercise training and dietary advice have shown favorable trends on reducing excessive weight gain, GDM, and macrosomia, but only three trials using dietary advice in GDM prevention have been published [31]. In these trials, low-glycemic diet was related to an average of 446 g lighter babies, but the evidence is still inconclusive due to small sample sizes and diverse outcomes in the trials [31]. In our study, the changes observed in dietary outcomes especially by 36–37 wk gestation may at least partly explain the between-group difference observed in birthweight of the newborns. On the other hand, time from the initiation of the dietary counseling (16–18 wk gestation onwards) to the measurement of GDM at 26–28 wk gestation may have been too short to produce changes in dietary habits and further to have an effect on development of GDM. Changes in both physical activity and diet were fairly similar as published in our pilot trial [15],[16]. Adherence to the lifestyle aims is a significant issue when considering effect of the intervention on the primary outcomes. Our definition for adherence included achievement of at least four dietary aims and/or the physical activity aim and the weight gain recommendation. Our analyses on adherence suggest that achievement of the aims of all these three components of the intervention (rather than only part of them) is associated with a lower risk for LGA and may be associated with lower risk of GDM.

Another possible reason for negative result concerning GDM prevention may be the risk group status of the women recruited to the trial. Since we included women with at least one GDM risk factor, most women had quite low risk for developing GDM. If we had included women with high risk of GDM, e.g., obese women or women with previous insulin-treated GDM, the results might have been different.

Our study did not show an increase in the incidence of adverse events or preterm birth in the intervention group. Thus, lifestyle counseling implemented by the nurses may be considered safe. Our counseling procedure has been shown to be feasible [15],[16], and it may be more applicable in maternity health care than interventions delivered by research nurses or other staff. In a study with individual randomization, not only statistical power, but also risk for contamination between trial arms would have been higher than in our cluster-randomized trial. The generalizability of our findings is higher than efficacy trials due to implementation in real-world instead of laboratory settings, although limited to women with no abnormal findings in OGTT during 8–12 wk gestation.

Limitations of our study also include the absence of late pregnancy measurement of maternal glucose intolerance, and owing to this, we were not able to assess maternal endpoints close to delivery, and thus high birthweight was used as a marker of longstanding glucose intolerance during pregnancy. Inaccuracy in birthweight, crown-heel length, and head circumference measurements in hospital is likely to be nondifferential, since the possibility of such errors was equal in both groups. Secondly, the differences between groups might have been even larger if this inaccuracy had not existed. An inevitable limitation is also that the women and the nurses in the usual care group could not be blinded for the purpose of the study, which may have resulted in changes in their health behavior or counseling practices.

### Conclusion

Evidence on the primary prevention of GDM and its consequences among women at risk but with normal glucose tolerance has been lacking. Our study has shown that lifestyle counseling is effective in decreasing newborns' birthweight among women at risk of GDM and producing behavioral change. We failed to find an effect on GDM diagnosed at 26–28 wk gestation or later, but the analyses performed among the adherent women suggest that favorable changes in behavior may decrease the risk of LGA offspring. Results from ongoing clinical trials [32],[33] may strengthen the evidence on the effectiveness of lifestyle modifications on maternal and fetal hyperglycemia and its consequences. The findings of our study emphasize counseling on the topics of physical activity, diet, and weight gain in maternity care especially for women at risk for GDM in order to prevent LGA newborns possibly causing problems in delivery, and both the mother's and the child's later weight development.

## Supporting Information

Alternative Language Abstract S1 French translation of the abstract.
(DOC)

Alternative Language Abstract S2 Italian translation of the abstract.
(DOC)

Alternative Language Abstract S3 Portuguese translation of the abstract.
(DOC)

Alternative Language Abstract S4 Spanish translation of the abstract.
(DOC)

Table S1 Inclusion criteria, pre-pregnancy BMI, education, smoking, parity and age by clusters in intervention and usual care areas.
(DOC)

Text S1 Protocol (previously published in [17]).
(PDF)

Text S2 CONSORT checklist.
(DOC)

## Abbreviations

BMI: body mass index
CI: confidence interval
GDM: gestational diabetes mellitus
LGA: large for gestational age
OGTT: oral glucose tolerance test
SD: standard deviation
SGA: small for gestational age
