# Supplementary material for: Primary Prevention of Gestational Diabetes Mellitus and Large-for-Gestational-Age Newborns by Lifestyle Counseling: A Cluster-Randomized Controlled Trial
Source: PLoS Med. 2011 May 17;8(5):e1001036. doi: 10.1371/journal.pmed.1001036 (PMC3096610; doi:10.1371/journal.pmed.1001036)
Supplement: Alternative Language Abstract S1 — French translation of the abstract. (DOC) [file pmed.1001036.s001.doc]

**Prévention primaire du diabète gestationnel et des nouveau-nés trop gros pour le terme via des conseils en style de vie - essai clinique randomisé.**

Objectif : étudier si le diabète gestationnel (GDM - gestational diabetes mellitus) ou le poids à la naissance élevé du nouveau-né peut être évité grâce à des conseils en style de vie.

Conception et méthodes de recherche : essai clinique randomisé réalisé dans 14 municipalités en Finlande, où 2271 femmes enceintes de 8 à 12 semaines ont été examinées par **épreuve de tolérance orale** au glucose (OGTT - oral glucose tolerance test). Les femmes en euglycémie (N=399) portant au moins un facteur de risque de GDM (indice de masse corporelle (IMC) ≥ 25 kg/m², l’intolérance au glucose ou la macrosomie du nouveau-né (≥ 4500 g) au stade de grossesse précoce, l’historique diabétique de la famille, âge ≥ 40 ans) étaient inclus. L’intervention a induit des conseils individuels soutenus sur l’activité physique, le régime alimentaire et la prise de poids lors de cinq visites prénatales. Les résultats primaires ont été l’incidence de GDM (santé maternelle) et le poids à la naissance des nouveau-nés (santé néonatale). Les résultats secondaires ont été la prise de poids maternelle et le besoin de traitement d’insuline pendant la grossesse. L’adhésion à l’intervention a été évaluée sur la base des changements de l’activité physique et du régime alimentaire.

**Résultats :** 15,8 % (34/216) de femmes dans le groupe d’intervention et 12,4% (22/179) dans le groupe de soins habituels ont été traitées par GDM (effet d’ampleur absolu 1.36, 95 % CI 0.71-2.62, p=0,36). Le poids à la naissance était plus faible dans le groupe d’intervention que dans le groupe de soins habituels (effet d’ampleur absolu-133 g, 95 % CI -231 à -35, p=0,008) comme l’était la proportion de nouveau-nés trop gros pour le terme (LGA - large for gestational age) (26/216, 12,1 % contre 34/179, 19,7 %, p=0,042). Les femmes dans le groupe d’intervention ont augmenté la prise de fibres alimentaires et d’acides gras polyinsaturés, diminué la prise d’acides gras saturés et de saccharose et ont eu tendance à voir leur niveau de MET minutes/semaine réduit dans des proportions inférieures pour une activité d’intensité au moins modérée que les femmes dans le groupe de soins habituels. Les femmes dépendant du groupe d’intervention (N=55/229) avaient moins de GDM (27,3 % contre 33,0 %, p=0,43) et de nouveau-nés LGA (7,3 % vs 19,5 %, p=0,03) comparé aux femmes dans le groupe de soins intensifs.

**Conclusions**: l’intervention a été efficace pour contrôler le poids à la naissance des nouveau-nés, mais a échoué à avoir un effet sur le GDM.
