# Supplementary material for: Primary Prevention of Gestational Diabetes Mellitus and Large-for-Gestational-Age Newborns by Lifestyle Counseling: A Cluster-Randomized Controlled Trial
Source: PLoS Med. 2011 May 17;8(5):e1001036. doi: 10.1371/journal.pmed.1001036 (PMC3096610; doi:10.1371/journal.pmed.1001036)
Supplement: Alternative Language Abstract S2 — Italian translation of the abstract. (DOC) [file pmed.1001036.s002.doc]

**Prevenzione primaria del diabete mellito gestazionale e del peso maggiore per l'età gestazionale nei neonati tramite consulenza sullo stile di vita: trial randomizzato controllato in cluster**

Obiettivo: Esaminare se il diabete mellito gestazionale (GDM) o l'elevato peso alla nascita dei neonati può essere prevenuto tramite consulenza sullo stile di vita.

Intento della ricerca e metodi: Un trial randomizzato controllato in cluster in 14 comuni della Finlandia, in cui è stato effettuato lo screening di 2271 donne con un test da carico orale di glucosio (OGTT) a 8-12 settimane di gestazione. Sono state incluse nello studio donne euglicemiche (N=399) con almeno un fattore di rischio GDM (indice di massa corporea (BMI) ≥ 25 kg/m², intolleranza al glucosio o macrosomia del neonato (≥ 4500 g) in una gravidanza precedente, anamnesi familiare di diabete, età ≥ 40 anni). L'intervento comprendeva una consulenza individuale intensificata su attività fisica, dieta e aumento di peso in cinque visite prenatali. Gli esiti primari erano l'incidenza di GDM (esito materno) e peso alla nascita dei neonati (esito neonatale). Gli esiti secondari erano l'aumento di peso materno e la necessità di trattamento con insulina durante la gravidanza. L'adesione all'intervento è stato valutato sulla base dei cambiamenti nell'attività fisica e nella dieta.

**Risultati**: Il 15,8% (34/216) delle donne nel gruppo di intervento e il 12,4% (22/179) nel gruppo trattato tradizionalmente ha presentato GDM (misure di effetto assoluto 1,36, 95% CI 0,71-2,62, p=0,36). Il peso alla nascita era inferiore nel gruppo di intervento rispetto al gruppo trattato tradizionalmente (misure di effetto assoluto -133 g, 95% CI da -231 a -35, p=0,008), in quanto neonati di peso maggiore per l'età gestazionale (LGA) (26/216, 12,1% contro 34/179, 19,7%, p=0,042). Le donne nel gruppo di intervento hanno aumentato il consumo di fibre alimentari e acidi grassi polinsaturi, hanno ridotto l'assunzione di acidi grassi saturi e saccarosio ed hanno riportato una lieve tendenza a diminuire in MET/minuti alla settimana per attività di intensità almeno moderata rispetto alle donne del gruppo trattato tradizionalmente. Le donne del gruppo di intervento (N=55/229) hanno avuto neonati con GDM inferiore (27,3% contro 33,0%, p=0,43) ed LGA ridotto (7,3% contro 19,5%, p=0,03) rispetto alle donne del gruppo trattato tradizionalmente.

**Conclusioni**: L'intervento è stato efficace nel controllo del peso alla nascita dei neonati, ma non è riuscito a riscontrare un effetto sul GDM.
