# Supplementary material for: Primary Prevention of Gestational Diabetes Mellitus and Large-for-Gestational-Age Newborns by Lifestyle Counseling: A Cluster-Randomized Controlled Trial
Source: PLoS Med. 2011 May 17;8(5):e1001036. doi: 10.1371/journal.pmed.1001036 (PMC3096610; doi:10.1371/journal.pmed.1001036)
Supplement: Alternative Language Abstract S3 — Portuguese translation of the abstract. (DOC) [file pmed.1001036.s003.doc]

**Prevenção primária da diabetes mellitus gestacional (DMG) e recém-nascidos grandes para a idade gestacional por orientação de estilo de vida – um estudo controlado aleatorizado, por agrupamentos**

Objetivo: Para examinar se a diabetes mellitus gestacional (DMG) ou o alto peso de recém-nascidos pode ser prevenido por orientação de estilo de vida.

Projeto e métodos de pesquisa: Um estudo aleatorizado por agrupamentos em 14 cidades na Finlândia, onde 2.271 mulheres foram detectadas através de teste de tolerância à glicose oral (OGTT) em gestação de 8 a 12 semanas. Foram incluídas mulheres euglicêmicas (N=399) com pelo menos um fator de risco da DMG (índice de massa corporal (BMI) ≥ 25 kg/m², intolerância à glicose ou macrossomia em recém-nascidos (≥ 4500 g) em gestação precoce, histórico de diabetes na família, ≥ 40 anos). A intervenção incluiu orientação individual intensificada em atividade física, dieta e ganho de peso em cinco consultas de pré-natal. Os resultados primários incluíram incidência de DMG (resultado maternal) e peso de nascimento de recém-nascidos (resultado neonatal). Os resultados secundários incluíram ganho de peso maternal e a necessidade de tratamento com insulina durante a gravidez. A adesão à intervenção foi avaliada com base em mudanças na atividade física e dieta.

**Resultados**: 15,8% (34/216) das mulheres no grupo de intervenção e 12,4% (22/179) no grupo de atendimento normal tinham DMG (tamanho de efeito absoluto 1.36, 95% CI 0,71-2,62, p=0,36). O peso de nascimento foi menor na intervenção do que no grupo de atendimento normal (tamanho de efeito absoluto -133 g, 95% CI -231 a -35, p=0,008) como foi a proporção de recém-nascidos grandes para a idade gestacional (26/216, 12,1% vs 34/179, 19,7%, p=0,042). As mulheres no grupo de intervenção aumentaram a ingestão de fibra alimentar e ácidos graxos polinsaturados, diminuíram a ingestão de ácidos graxos saturados e sacarose e apresentaram tendência a uma redução menor em METminutos/semana para, pelo menos, atividade de intensidade moderada do que as mulheres no grupos de atendimento normal. As mulheres que aderiram no grupo de intervenção (N=55/229) tiveram menos DMG (27,3% vs 33,0%, p=0,43) e recém-nascidos grandes para a idade gestacional (7,3% vs 19,5%, p=0,03) em comparação às mulheres do grupo de atendimento normal.

**Conclusões**: A intervenção foi efetiva no controle do peso de nascimento dos recém-nascidos, porém não foi eficaz na identificação de um efeito sobre a DMG.
