# Supplementary material for: Primary Prevention of Gestational Diabetes Mellitus and Large-for-Gestational-Age Newborns by Lifestyle Counseling: A Cluster-Randomized Controlled Trial
Source: PLoS Med. 2011 May 17;8(5):e1001036. doi: 10.1371/journal.pmed.1001036 (PMC3096610; doi:10.1371/journal.pmed.1001036)
Supplement: Alternative Language Abstract S4 — Spanish translation of the abstract. (DOC) [file pmed.1001036.s004.doc]

**Prevención primaria de la diabetes mellitus gestacional y los neonatos grandes para la edad gestacional a través del asesoramiento de estilo de vida - Estudio controlado aleatorizado en grupos.**

Objetivo: Examinar si la diabetes mellitus gestacional (DMG) o el alto peso al nacer de los neonatos puede prevenirse mediante asesoramiento de estilo de vida.

Diseño y métodos del estudio: Un ensayo aleatorizado en grupos en 14 municipios de Finlandia, con selección de 2.271 mujeres mediante prueba oral de tolerancia a la glucosa (POTG) en las 8-12 semanas de gestación. Se incluyó a mujeres euglucémicas (N=399) con al menos un factor de riesgo de DMG (índice de masa corporal (IMC) ≥ 25 kg/m², intolerancia a la glucosa o macrosomía del neonato (≥ 4.500 g) en cualquier embarazo anterior, historia familiar de diabetes, edad ≥ 40 años). La intervención incluyó asesoramiento intensificado individual sobre la actividad física, la dieta y el aumento de peso en cinco visitas prenatales. Los resultados principales fueron la incidencia de la DMG (resultado para la madre) y el peso al nacer del neonato (resultado para el neonato). Los resultados secundarios fueron el aumento de peso de la madre y la necesidad de tratamiento con insulina durante el embarazo. El cumplimiento de la intervención fue evaluado a partir de los cambios en la actividad física y la dieta.

**Resultados**: El 15,8% (34/216) de las mujeres del grupo de intervención y 12,4% (22/179) del grupo que recibió la asistencia habitual, presentaron DMG (magnitud de efecto absoluto 1,36, 95% CI 0,71-2,62, p=0,36). El peso al nacer fue más bajo en la intervención que en el grupo que recibió la asistencia convencional (magnitud de efecto absoluto -133 g, 95% CI -231 a -35, p=0,008), al igual que la proporción de neonatos grandes para la edad gestacional (GEG) (26/216, 12,1% vs 34/179, 19,7%, p=0,042). Las mujeres del grupo de intervención incrementaron su ingesta de fibra dietética y ácidos grasos poliinsaturados, redujeron la ingesta de ácidos grasos saturados y sacarosa y presentaron tendencia hacia una mayor reducción de los MET-minuto/semana durante la actividad de intensidad al menos moderada que las mujeres del grupo que recibió la asistencia convencional. Las mujeres del grupo de intervención que cumplieron las recomendaciones (N=55/229). presentaron DMG en menor grado (27,3% frente a 33,0%, p=0,43) y neonatos GEG (7,3% frente a 19,5%, p=0,03) con respecto a las mujeres del grupo que recibió la asistencia convencional.

**Conclusiones**: La intervención resultó eficaz a la hora de controlar el peso al nacer de los neonatos, pero no consiguió obtener efectos en cuanto a la DMG.
