# Supplementary material for: Primary Prevention of Gestational Diabetes Mellitus and Large-for-Gestational-Age Newborns by Lifestyle Counseling: A Cluster-Randomized Controlled Trial
Source: PLoS Med. 2011 May 17;8(5):e1001036. doi: 10.1371/journal.pmed.1001036 (PMC3096610; doi:10.1371/journal.pmed.1001036)
Supplement: Table S1 — Inclusion criteria, pre-pregnancy BMI, education, smoking, parity and age by clusters in intervention and usual care areas. (DOC) [file pmed.1001036.s005.doc]

| Table S1. Inclusion criteria, pre-pregnancy BMI, education, smoking, parity and age by clusters in intervention and usual care areas. | **Intervention areas** | | | | | | | **Usual care areas** | | | | | | |
| --- | --- | --- | --- | --- | --- | --- | --- | --- | --- | --- | --- | --- | --- | --- |
|  | 1 | 2 | 3 | 4 | 5 | 6 | 7 | 8 | 9 | 10 | 11 | 12 | 13 | 14 |
| Frequency of inclusion criteria^1^ | N (%) | N (%) | N (%) | N (%) | N (%) | N (%) | N (%) | N (%) | N (%) | N (%) | N (%) | N (%) | N (%) | N (%) |
| 1 | 29  (66) | 25  (76) | 28  (61) | 26  (81) | 17  (43) | 13  (62) | 63  (63) | 15  (52) | 23  (68) | 57  (71) | 31  (51) | 10  (63) | 28  (70) | 13  (57) |
| 2 | 12  (27) | 7  (21) | 17  (37) | 5  (16) | 19  (48) | 8  (38) | 34  (34) | 10  (35) | 11  (32) | 18  (23) | 27  (44) | 4  (25) | 10  (25) | 7  (30) |
| 3-4 | 3  (7) | 1  (3) | 1  (2) | 1  (3) | 4  (10) | 0  (0) | 3  (3) | 4  (14) | 0  (0) | 5  (6) | 3  (5) | 2  (13) | 2  (5) | 3  (13) |
| Pre-pregnancy BMI (kg/m2) |  |  |  |  |  |  |  |  |  |  |  |  |  |  |
| < 20 | 4  (9) | 1  (6) | 1  (3) | 3  (10) | 1  (2) | 0  (0) | 3  (3) | 2  (12) | 0  (0) | 1  (2) | 3  (5) | 1  (8) | 1  (3) | 2  (9) |
| 20-24.9 | 16  (36) | 8  (44) | 15  (48) | 9  (29) | 10  (24) | 7  (35) | 24  (26) | 5  (29) | 3  (20) | 20  (33) | 19  (30) | 4  (31) | 14  (45) | 4  (17) |
| ≥ 25 | 25  (56) | 9  (50) | 15  (48) | 19  (61) | 31  (74) | 13  (65) | 65  (71) | 10  (59) | 12  (80) | 40  (66) | 42  (66) | 8  (62) | 16  (52) | 17  (74) |
| Education^2^ |  |  |  |  |  |  |  |  |  |  |  |  |  |  |
| High | 6  (15) | 8  (26) | 12  (27) | 12  (39) | 10  (26) | 9  (45) | 20  (22) | 0  (0) | 6  (19) | 14  (18) | 11  (17) | 1  (7) | 11  (27) | 5  (25) |
| Medium | 18  (46) | 11  (36) | 14  (32) | 12  (39) | 16  (42) | 9  (45) | 45  (48) | 14  (61) | 11  (36) | 33  (43) | 26  (41) | 5  (33) | 18  (44) | 7  (35) |
| Low | 15  (39) | 12  (39) | 18  (41) | 7  (23) | 12  (32) | 2  (10) | 28  (30) | 9  (39) | 14  (45) | 29  (38) | 27  (42) | 9  (60) | 12  (29) | 8  (40) |
| Smokers^3^ | 4  (11) | 3  (13) | 8  (20) | 2  (8) | 4  (12) | 3  (16) | 17  (21) | 1  (5) | 6  (27) | 5  (8) | 5  (9) | 0  (0) | 7  (21) | 0  (0) |
| Primiparous | 13  (31) | 10  (48) | 8  (42) | 20  (65) | 23  (55) | 4  (20) | 39  (42) | 9  (53) | 9  (53) | 26  (41) | 30  (47) | 2  (15) | 14  (44) | 5  (22) |
| Age (mean, SD) | 29.3  5.0 | 29.5  4.8 | 29.7  4.8 | 29.7  3.8 | 29.3  5.0 | 29.4  4.4 | 29.3  5.1 | 27.6  3.5 | 30.4  5.1 | 30.1  4.2 | 29.6  4.6 | 27.5  6.1 | 31.2  4.7 | 29.6  4.9 |

1) Inclusion criteria: BMI ≥25 kg /m^2^, glucose intolerance/newborn’s macrosomia (≥ 4500 g) in any earlier pregnancy, family history of diabetes, age ≥ 40 years

2) High: university degree; Medium: polytechnic education; Low: basic or secondary education

3) Smoking before or during pregnancy
