# Supplementary material for: Primary Prevention of Gestational Diabetes Mellitus and Large-for-Gestational-Age Newborns by Lifestyle Counseling: A Cluster-Randomized Controlled Trial
Source: PLoS Med. 2011 May 17;8(5):e1001036. doi: 10.1371/journal.pmed.1001036 (PMC3096610; doi:10.1371/journal.pmed.1001036)
Supplement: Text S1 — Protocol (previously published in [17]). (PDF) [file pmed.1001036.s006.pdf]

Luoto et al. BMC Pregnancy and Childbirth 2010, 10:39
http://www.biomedcentral.com/1471-2393/10/39

BMC Pregnancy & Childbirth

STUDY PROTOCOL Open Access

Prevention of Gestational Diabetes: Design of a Cluster-Randomized Controlled Trial and One-Year Follow-Up

Riitta M Luoto[1,2*], Tarja I Kinnunen[3], Minna Aittasalo[1], Katriina Ojala[1], Kirsi Mansikkamäki[1], Erja Toropainen[1], Päivi Kolu[1], Tommi Vasankari[1,2]

Abstract

Background: Annual prevalence of gestational diabetes mellitus (GDM) is 12.5% among Finnish pregnant women. The prevalence is expected to rise with the increasing overweight among women before pregnancy. Physical activity and diet are both known to have favourable effects on insulin resistance and possibly on the risk of GDM. We aimed to investigate, whether GDM can be prevented by counseling on diet, physical activity and gestational weight gain during pregnancy.

Methods/Design: A cluster-randomized controlled trial was conducted in 14 municipalities in the southern part of Finland. Pairwise randomization was performed in order to take into account socioeconomic differences. Recruited women were at 8-12 weeks' gestation and fulfilled at least one of the following criteria: body mass index ≥ 25 kg/m^2, history of earlier gestational glucose intolerance or macrosomic newborn (> 4500 g), age ≥ 40 years, first or second degree relative with history of type 1 or 2 diabetes. Main exclusion criterion was pathological oral glucose tolerance test (OGTT) at 8-12 weeks' gestation. The trial included one counseling session on physical activity at 8-12 weeks' gestation and one for diet at 16-18 weeks' gestation, and three to four booster sessions during other routine visits. In the control clinics women received usual care. Information on height, weight gain and other gestational factors was obtained from maternity cards. Physical activity, dietary intake and quality of life were followed by questionnaires during pregnancy and at 1-year postpartum. Blood samples for lipid status, hormones, insulin and OGTT were taken at 8-12 and 26-28 weeks' gestation and 1 year postpartum. Workability and return to work were elicited by a questionnaire at 1- year postpartum. Linkage to the national birth register of years 2007-2009 will provide information on perinatal complications and GDM incidence among the non-participants of the study. Cost-effectiveness evaluation will be based on quality-adjusted life years. This study has received ethical approval from the Ethical board of Pirkanmaa Hospital District.

Discussion: The study will provide information on the effectiveness and cost-effectiveness of gestational physical activity and dietary counseling on prevention of GDM in a risk group of women. Also information on the prevalence of GDM and postpartum metabolic syndrome will be gained. Results on maintaining the possible health behaviour changes are important in order to prevent chronic diseases such as cardiovascular disease and diabetes.

Trial registration: The trial is registered ISRCTN 33885819

BioMed Central

© 2010 Luoto et al; licensee BioMed Central Ltd. This is an Open Access article distributed under the terms of the Creative Commons Attribution License (http://creativecommons.org/licenses/by/2.0), which permits unrestricted use, distribution, and reproduction in any medium, provided the original work is properly cited.

Background

Gestational diabetes mellitus (GDM) is defined as a type of diabetes firstly diagnosed during pregnancy [1,2]. Insulin sensitivity usually decreases towards the end of pregnancy and is therefore partly physiological, but among part of pregnant women it results to glucose intolerance and GDM [3]. Prevalence estimates vary, usually approximately 5% of pregnant women develop GDM [4]. In Finland the average prevalence of GDM is 12.5%, but varies from 6 to 25% between different hospital districts, at least partly due to different diagnostic criteria [5].

Although glucose metabolism usually normalises after delivery, majority of women with GDM have an increased risk of later diabetes mellitus or impaired glucose tolerance and possibly also of metabolic syndrome [6-9]. In a Danish sample (N = 481), the prevalence of metabolic syndrome approximately 10 years after pregnancy was three times as high in women with prior diet-treated GDM as in 1000 age-matched population-based control subjects [10]. Although the prevalence of metabolic syndrome after one year is lower than ten years after delivery, women with GDM risk have been reported as forerunners of maternal and childhood obesity [8]. GDM and overall obesity among women reflects also to children's weight development. Women with GDM have more often macrosomic newborns (birth weight ≥ 4000 g) and the related health problems than other women [11]. Intrauterine hyperglycaemia may contribute to the pathogenesis of offspring overweight and metabolic syndrome later in life [12].

The most important risk factors of GDM are high maternal age, family history of type 2 diabetes and overweight before pregnancy [13] and GDM or glucose intolerance in previous pregnancies [7]. There is also some evidence that excessive gestational weight gain, high intake of saturated fat and low intake of polyunsaturated fat may increase the risk of GDM [4,14-17]. On the other hand, physical activity improves glucose tolerance and insulin sensitivity in pregnant women [18]. Physical activity before or during pregnancy is also associated with reduced risk of GDM [19-21]. In the case-control study by Dempsey et al. [22] women, who were engaged in physical activity had almost 50% reduction in risk of GDM compared with inactive women.

Lifestyle modifications have been shown valuable adjunctive therapy in the treatment of GDM [23]. Moreover, there is encouraging evidence on the impact of lifestyle interventions to prevent the progression of GDM to type 2 diabetes in women with history of GDM [24]. The small trials by Mottola et al. [25] and Hui et al [26] showed promising results in preventing GDM with nutrition and physical activity modification. In our own pilot study (n = 105), pregnant women who had intensified dietary and physical activity counseling at routine maternity clinic visits were able to change their diet to favourable direction and to maintain at least moderate physical activity more often than the women with routine counseling [27,28]. Interestingly, there were no high birth weight newborns in the intervention group, but eight of them (15%) in the control group, suggesting that the intervention may have had beneficial effect on maternal glucose tolerance.

The evidence for the effect of lifestyle modification in preventing GDM will accumulate in the future from ongoing studies by Chasan-Taber et al [29] and Oostdam et al [30] and from our study presented here. The *primary objective* of our trial is to show whether individual counseling on physical activity, diet and gestational weight gain can have preventive effect on the development of GDM. *Secondary objectives of our trial* are to evaluate maternal and child weight development, maternal metabolic changes, and changes in maternal physical activity and dietary habits and quality of life. Cost-effectiveness of the trial is evaluated since the prevention of GDM is important in order to reduce direct health care costs, such as hospital days and surgical procedures and indirect costs due to sick leave during pregnancy. To date no cost-effectiveness studies have been published on the prevention of GDM. The purpose of this paper is to describe the design and the methods of a cluster-randomized controlled trial (RCT) to prevent GDM and its 1-year follow-up.

Methods/Design

Study design, setting and group allocation

The RCT was conducted in primary health care maternity clinics in Pirkanmaa region, situated south-western part of Finland (Figure 1). The prevalence of GDM was 17% in Pirkanmaa region in 2004[5]. Municipalities in Pirkanmaa region with at least 70 annual deliveries were contacted and recruited to the study (N = 14). The city of Tampere, with more than 200.000 inhabitants and 23 maternity clinics, was the biggest of these municipalities. However, it was the only municipality restricting the number of participating maternity clinics to one due to the extra workload accumulated to the nurses from the study arrangements and counseling. All clinics from the other municipalities took part in the study.

For the cluster randomisation, the municipalities were arranged into matched pairs and within each pair they were randomized to an trial and a control municipality. The pair-matching was done with regard to number of births, size and socio-economic level of the population, estimated incidence of GDM and whether the clinic situated in rural or urban area. Municipalities were

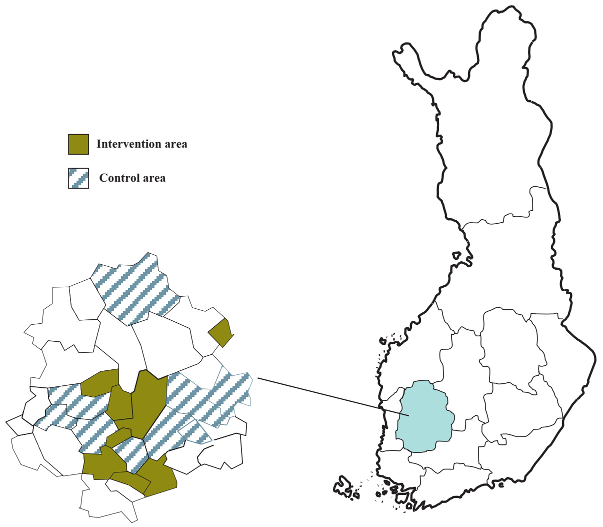

Figure 1 The trial and the control municipalities in Pirkanmaa area in south-western Finland.

randomized rather than individual clinics, nurses or pregnant women to avoid contamination.

In Finland, maternity health care is provided by the municipalities and funded through public taxation. The visits are free of charge for women. Almost all pregnant women (99.7%) utilize municipal maternity care services, although there are private services available as well [5,31]. Currently, first-time pregnant women are recommended to visit a nurse 11-15 times and a physician three times during pregnancy [31,32]. The recommended number of visits is lower (7-11) during subsequent pregnancies. The first visit to maternity clinics usually takes place when women are at approximately 8 to 12 weeks' gestation. The spouses are encouraged to attend all visits.

Recruitment of study population

In total, 23 nurses from the trial clinics and 30 nurses from the control clinics participated in the implementation of the study. The nurses recruited pregnant women when they contacted the maternity clinic for the first time by telephone (up to 12 weeks' gestation). The women who were interested in participating in trial received an invitation letter and the informed consent form by mail. The eligible women willing to participate signed the informed consent during the first maternity visit. If the women were not eligible or not willing to participate, they were asked to complete a baseline questionnaire and another informed consent for later linkage to the birth registry information.

The nurses used structured form to keep record on the women invited and eligible to the study and of those eligible or not wanting to participate. The form also included space for entering the reasons for dropping out. Recruitment was planned to continue until at least 30 women were enrolled in each municipality. All participants of the trial including the non-participants who were willing to fill the baseline questionnaire, were included in the follow-up study. Ethical approval for this study was obtained from the Ethical board of Pirkanmaa Hospital District (Reference number R06230, 19.1.2007).

Inclusion and exclusion criteria

Inclusion criteria

Pregnant women were eligible for the study if they had at least one of the following risk factors: BMI ≥ 25 kg/m^2, GDM or any signs of glucose intolerance or macrosomic newborn (≥ 4500 g) in any earlier pregnancy, type 1 or 2 diabetes in first or second grade relatives or age ≥ 40 years.

Exclusion criteria

Women were excluded if they had at least one of the following: a pathological value in the baseline oral glucose tolerance test (OGTT) at 8-12 weeks' gestation (blood glucose > 5.3 mmol/l at fasting, > 10.0 mmol/l at 1-hour or >8.6 mmol/l at 2-hour), pre-pregnant type 1 or 2 diabetes, inability to speak Finnish, age < 18 years, twin pregnancy, physical restriction preventing from physical activity, substance abuse, treatment or clinical history of psychiatric illness.

Intervention

The trial will continue from the first maternity clinic visit (at 8-12 weeks' gestation) until 37 weeks' gestation (Figure 2). The recommendations for gestational weight gain are discussed and the primary physical activity counseling takes place at the first visit. The primary dietary counseling session is implemented during the visit at 16-18 weeks' gestation. Both physical activity and dietary counseling are boostered at subsequent visits; physical activity at four and diet at three visits (Figure 2).

Both physical activity and dietary counseling are based on the model of Laitakari and Asikainen [33] which incorporates two central behavioral models, PRECEDE-PROCEED [34] and Stages of Change ([35]. The model has been found applicable in occupational health care [36] and in the pilot study on preventing excessive gestational weight gain [27,28]. The allocated time regarding both physical activity and dietary counseling was 20-30 minutes for primary counseling sessions and 10-15 minutes for each of the booster sessions [27].

Counseling on gestational weight gain

The recommendations for total gestational weight gain were 12.0-18.0 kg for women with pre-pregnancy BMI 18.5-19.9 kg/m^2, 11.5-16.0 kg for women with BMI 20.0-26.0 kg/m^2 and 7.0-11.5 kg for women with BMI ≥ 26.0 kg/m^2 [37]. These recommendations were

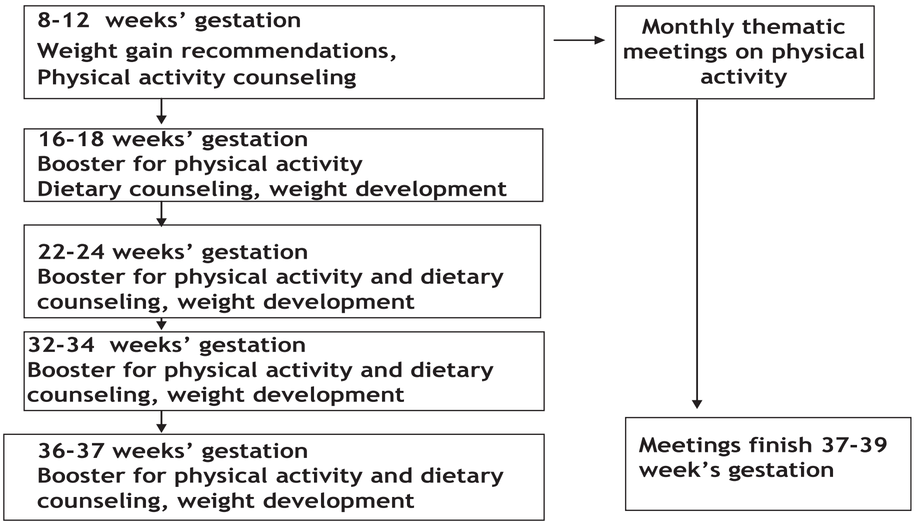

Figure 2 Timing of the counseling in the trial clinics.

introduced at the first maternity clinic visit. In order to help the participant to reach her BMI-specific recommendation, her rate of weight gain was monitored until the end of pregnancy by using a BMI-specific weight gain chart [38]. This chart was included in the participant's own follow-up notebook. Allocated time for discussion on the weight gain recommendations and weight monitoring was 5 minutes at each of the five visits.

Physical activity counseling

According to current American [39,40] and Canadian guidelines [41] the general physical activity recommendation for health [42] applies to pregnant women without medical or obstetric complications. This has been restated in the most recent physical activity recommendation updated by the Physical Activity Guidelines Advisory Committee [43]. The general recommendation for fitness [44] is also valid during pregnancy among women, who have been regular exercisers before pregnancy and who have uncomplicated pregnancy [40]. Borg's [45,46] visual scale of perceived exertion (RPE) including ratings 6-20 is suggested for intensity assessment. The objectives of the physical activity counseling were:

1) To increase leisure time physical activity (LTPA) of those pregnant women, who are not fulfilling the recommendations, to the recommended level for health.

2) To maintain LTPA of those pregnant women, who are already fulfilling the recommendation for health.

3) To maintain or adjust LTPA of those pregnant women, who are already fulfilling the recommendation for fitness.

The structure and the topics of the physical activity counseling sessions were guided by the counseling card, which was filled in for each participant at each session. At the primary session, the participant's current leisure-time physical activity (LTPA) and her need and opportunities for LTPA were assessed and the benefits and limitations on LTPA were discussed with the help of a take-home leaflet. The nurse and participant agreed on a weekly action plan including LTPA modes and their frequency, duration and intensity, which was based on RPE ratings 6-20 [45,46].

The minimum weekly LTPA dose entered progressively in the action plan was 800 MET (multiples of resting metabolic equivalents) minutes. Thus is in line with Haskell et al [47] suggesting the minimum of 450 to 750 weekly MET minutes for health. Nurses calculated MET minutes from the action plans by multiplying the weekly minutes and the MET value of each LTPA mode and by summing up the numbers. Also light-intensity LTPA (MET value 3) was included to the plan due to participants' different LTPA backgrounds. RPE 6-11 equaled three METs, 12-14 five METs and 14-20 seven METs [41,44,48,49]. Compliance with the action plan was monitored at the booster sessions with a LTPA log, which was part of the participant's follow-up notebook. If the action plan was revised, also the weekly MET minutes were recalculated.

Monthly thematic meetings on physical activity

During the primary physical activity counseling visit the participants were offered an opportunity to participate monthly thematic meetings on physical activity including group exercise. The sessions were arranged after the working hours close to the maternity clinics and the women's living area. The purpose of the meetings was to support physical activity counseling by providing the participants social support for behaviour change and by introducing them various ways of being physically active.

Sessions consisted of five different themes on a non-stop basis, which were:

Theme 1. Physical activity is worthwhile during pregnancy: the benefits of physical activity, current physical activity recommendations during pregnancy.

Theme 2. Walking is pleasant and effective: putting technique and footwear in order.

Theme 3: More alteration and goals for walking: using pedometers and poles.

Theme 4: Preventing urinary incontinence: pelvic muscles and training.

Theme 5: Integrating physical activity to family life: physical activity after pregnancy.

The dates of all the sessions were informed during the primary counseling visit. The participants were to attend to the next meeting after their recruitment but were eventually able attend to every one of them during the pregnancy. The duration of each session was two hours: 30 minutes for getting acquainted, 30 minutes for the theoretical basis related to the theme and 1 hour for the group exercise related to the theme. In all the sessions RPE was used in assessing the intensity of exercise.

The sessions were instructed by the physiotherapists of local health care centres or private clinics. A week before each meeting a reminder of the forthcoming meeting was transmitted via short message service from the research institute to each participant. A week after the meeting the instructor contacted all the participants by telephone to encourage them to continue with their weekly action plans and to get feedback on the meeting from those who had attended. The instructors were trained and provided with all the material needed for the theoretical and practical parts of the thematic meetings. Also, they were paid for the time needed for the training, for the actual meetings and for making the telephone calls.

Dietary counseling

The aim of the dietary counseling was to help the participants to achieve a diet containing saturated fat ≤ 10%, polyunsaturated fat 5-10% and total fat 25-30% (includes saturated, monounsaturated, polyunsaturated and trans fatty acids) of total energy intake and fiber 25 to 35 g/day. This aim was selected based on the Finnish dietary recommendations[50], studies on the association of diet and development of GDM [15-17] study that was successful in preventing type 2 diabetes mellitus in Finland [51]. To achieve this, the dietary counseling focused on the objectives were:

1) To use vegetables, fruit and berries preferably at least 5 portions (400 g) a day.

2) To select mostly high fiber bread (≥ 6 g fiber/100 g) and other whole-meal products.

3) To select mostly fat-free or low-fat versions of milk and milk products (e.g. yoghurt, cheese, ice cream) and of meat and meat products.

4) To eat fish at least twice per week (excluding the fish species not recommended for pregnant women).

5) To use moderate amounts of soft table spreads on bread, oil-based salad dressing in salad and oil in cooking and baking.

6) To use seldom and only in small portion sizes foods high in fat.

7) To use seldom and only in small portion sizes snacks containing lots of sugar and/or fat (e.g. sweets, high-sugar drinks, cookies, ice cream, sweet and salty.

The nurse used a counseling card reminding her about the counseling topics and to which she was able to make remarks at each counseling session. At the primary counseling session, the nurse assessed the participant's current diet by using a check list containing the above mentioned objectives. The nurse asked the participant whether each of the objectives was fulfilled in her current diet either mostly, partly or not at all. The dietary recommendations were highlighted to the participants with the help of a leaflet on diet during pregnancy (Raskausajan liikunta ja ravitsemus, MLL & Sydänliitto), which was also given to the participant. Discussion included participant's needs for dietary changes, as well as her opportunities for and barriers to making the changes. The nurse encouraged the participant to maintain those objectives that were already fulfilled. Of the objectives that were not fulfilled, the participant selected 2-3 objectives which she wanted to improve in her diet. As a consequence of this discussion, individual objectives were set for the next visit (e.g. "to increase the intake of vegetables, fruit and berries from 2 to 4 portions/day" or "to decrease the intake of high-sugar snacks from 3 to 1 portion/day"). These objectives were written down in the follow-up note book. The participant was asked to keep weekly record whether she achieved her objectives or not. At each booster visit, the follow-up notebook was checked, the records were discussed and the objectives were revised if needed (e.g. set at higher level or changed to other objectives). The nurses also discussed about other dietary issues important for the participants (e.g. the special dietary restrictions during pregnancy).

Control group

The women in the control maternity clinics received routine care and no extra counseling beyond usual practices or group exercise were arranged. However, the routine maternity care includes some dietary and physical activity counseling as shown in our pilot study [27,28].

Non-participants

Women who were not willing to participate in the RCT were asked to complete the baseline questionnaire and to give their consent for linking their information to medical birth registry.

Outcome measurements

Outcome measurements are assessed at six times during pregnancy (8-12, 16-18, 22-24, 26-28, 32-34, 37-39 week's gestation) and twice postpartum (6 weeks and 1 year) (Figure 3, Table 1).

The primary maternal outcome of the trial is the proportion of women diagnosed with GDM, as assessed by oral glucose tolerance test during 26-28 week's gestation and the weight of the newborn adjusted for gestational age.

Secondary outcomes of the trial are gestational weight gain and need of insulin treatment during pregnancy determined at time of possible GDM diagnosis (earliest 26-28th week of pregnancy based on OGTT). Additional outcomes are changes in maternal physical activity (frequency and duration of total and domain-specific leisure time physical activity) and dietary habits (food choices related to the objectives of counselling, fibre intake (g/day) and proportions of saturated, polyunsaturated and total fat of total energy intake), maternal quality of life (profile of 15 health-related dimensions during pregnancy). Direct and indirect costs during pregnancy and the cost-effectiveness of the trial (based on QALYs) are calculated. Changes related to blood lipids (cholesterol, HDL-cholesterol, triglycerides, LDL-cholesterol, oxidized lipoproteins) and other biological markers of fat metabolics (IGF, IGFBp-3, C-peptide, leptin, adiponectin, para-oxonase) are determined both during pregnancy and one-year postpartum.

The outcomes of the 1-year follow-up study are maintenance of possible changes in physical activity and dietary habits, incidence of metabolic syndrome based on the International Diabetes Federation criteria [52], perceived workability, quality of life (RAND-36 scale), depression (Beck scale) and proportion of women returning to work after maternity leave.

Medical birth registry linkage includes information on perinatal outcomes (complications during pregnancy or delivery, such as pre-eclampsia, cesarean section rate and need for labour induction) and incidence of GDM among non-participants.

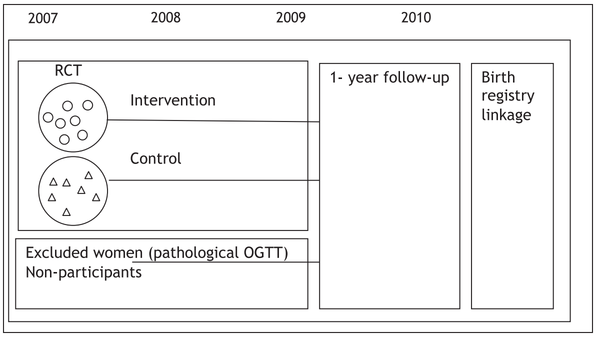

Figure 3 Design and timetable of the study: cluster-randomized controlled trial (RCT), 1-year follow-up and linkage to the medical birth registry.

Data collection

The timing of data collection for questionnaires, physical measurements and laboratory tests is described in detail in Table 1.

Questionnaires

Background information on participants was collected with the baseline questionnaire including information from socio-economic status, smoking, earlier weight development, use of medication and self-reported morbidity.

Physical activity habits were elicited at baseline, 36-37 weeks' gestation and 1-year postpartum. Baseline leisure-time physical activity (LTPA) during a typical week prior to the pregnancy and in the other questionnaires LTPA during a typical week of the previous three weeks was included. The degree of breathlessness (strong, some, none) was used to help the women to determine the intensity of their LTPA because there are indications that the meaning of intensity (hard, moderate, light) may be difficult to understand [53]. Validity and reliability of the physical activity questions were examined in a separate study [54].

Dietary habits were assessed by using a validated 181-item food frequency questionnaire (FFQ) [55]. At baseline, the women were asked questions about their diet during one month prior to the pregnancy, since their diet may have changed due to nausea or vomiting at the beginning of the pregnancy. In the follow-up, the women were asked questions about their diet during the previous month. The FFQ was completed during the OGTT during the trial and the follow-up, except for the second follow-up at 36-37 weeks' gestation when the FFQ was mailed to the participants' homes and it was returned to the nurse at the next visit or to the researchers by mail.

Quality of life evaluation was based on the 15-D, which is a validated instrument on quality of life [56] and visual analogue score (VAS) for perceived health. Perceived health has shown to be important factor in predicting functional capacity and health [57]. The 15 D is based on 15 separate items: ability to be physically active, vision, hearing, breathing, sleeping, eating, communicating, elimination, normal functions, mental health, symptoms and signs, depression, anxiety, vitality and sexuality. 15 D scale can be used as a profile including all variables or as a single index from zero to unity. In the 1-year follow-up

Table 1 Timing of questionnaires, maternal physical measurements and laboratory tests.

	Weeks' gestation						Postpartum follow-up	
	**8-12**	**16-18**	**22-24**	**26-28**	**32-34**	**36-37**	**6 weeks**	**1 year**
Questionnaires								
Background questions	X							
Physical activity	X			X		X		X
Food frequency	X			X		X		X
15 D quality of life	X			X		X	X	X
Adverse events		X	X	X	X	X		
Use of insulin therapy						X		X
Workability				X		X		X
Return to work								X
Physical measurements								
Height	X							
Weight	X	X	X	X	X	X	X	X
Blood pressure	X	X	X	X	X	X	X	X
Waist circumference								X
Laboratory tests								
Oral glucose tolerance test	X			X				X
Blood samples	X			X				X

study, also the Finnish version of RAND-36 [58] and Beck's depression scale [59] were used.

The use of insulin therapy was included in the questionnaire at 36-37 weeks' gestation.

Workability and factors related to return to work were elicited in the 1-year follow-up questionnaire. The workability questions were adopted from workability index developed in the Finnish Institute of Occupational Health. The index has been shown to predict long-term sick leave among young employees [60].

Physical measurements

Maternal anthropometric measures. Information on maternal anthropometric measurements were obtained from the standard maternity card. Pre-pregnancy weight was self-reported.The nurses measured body weight of the women at each study visit during pregnancy and height at the first visit. Total gestational weight gain was calculated based on the pre-pregnancy weight and last measured weight during pregnancy. One year postpartum the women were invited to local maternity clinics or to the UKK institute for weight, waist circumference and blood pressure measurements in order to determine the incidence of metabolic syndrome.

Foetal anthropometric measures. Weight of the newborn and gestational age was obtained from medical records of the delivery hospital or from the maternity card. Details related to delivery and perinatal complications will be received from medical birth registry.

Laboratory tests

Blood sample analyses included determination of lipids (cholesterol, HDL-cholesterol, triglycerides, LDL-cholesterol, oxidized lipoproteins) and other biological markers (IGF, IGFBp-3, C-peptide, leptin, adiponectin, para-oxonase, insulin) (Table 1). Medical laboratory technologists from the UKK Institute or from the Centre for Laboratory Medicine in Pirkanmaa hospital district took the blood samples (4 × 10 ml) and performed the OGTT for all participating pregnant women. Specimens are analyzed in analysed at Clarke-Hilakivi's laboratory at Georgetown University, Washington DC, USA (IGF-1, IGFBP-3, estradiol, progesterone, C-peptide) and in MCA research laboratory, Turku (leptin, adiponectin, insulin, lipids) and at UKK Institute.

OGTT. OGTT was taken at 8-12 and 26-28 weeks' gestation and 1 year postpartum. Analyses were performed at UKK Institute. OGTT has been shown to be the optimal test for insulin sensitivity during pregnancy [61]. In Finnish health care OGTT is performed for women at risk of GDM 26-28 weeks' gestation, because of the expected benefits of prevention of adverse pregnancy outcome related to GDM. We followed the same timing and conveyed OGTT results to nurses who were in charge for possible further treatment. The standard OGTT was performed by giving Glukodyn^{R}, including 75 g glucose in 330 ml water after overnight fasting (8 to 14 hours). The Finnish GDM criteria were used to identify participants with GDM [62,63]. Incremental and total area under curve (AUC) was also calculated based on the blood glucose values at 0, 1 and 2 hours after glucose ingestion. AUC can be used as a measure of glucose exposure and it gives a measure of how much and for how long glucose stays in

the blood. A long, low concentration exposure may be as important as shorter but higher concentration. Total AUC is a descriptive factor related to basal blood glucose value, whereas incremental AUC more accurately describe glycemic response to foods [64].

Adverse events

Adverse events related to recent physical activity were asked by the nurses at four visits with a structured form. The adverse events included were classified as warning signs for exercise termination by ACOG [39], e.g. vaginal bleeding, major contractions, dizziness, headache, chest pain and muscle weakness.

Cost-effectiveness

Total costs of the trial are calculated by summing up all direct costs and dividing the sum by the number of participants. Cost-effectiveness analysis includes differences in direct and indirect costs between the trial and the control groups. Direct costs include all consultations within the health care sector both in specialized and non-specialized care during the trial period (from baseline examinations until delivery). Hospital days, medications and laboratory cost are also included. Costs for the pregnant women include medications and travelling. Trial costs include trial materials, training the nurses for the counseling and the time used for implementation of the counselling and data collection at the routine maternity care visits. Indirect costs, like productivity loss includes absenteeism from work based on the number of days on sick leave during pregnancy. The evaluation of health outcomes is based on quality adjusted life years (QALY). The QALY is based on the number of years of life that would be added by the trial[65].

Process evaluation

The data collected for the process evaluation include e.g. the number of participating women in each clinic, participation rate among all invited women, drop out rate, number of returned questionnaires in the study and participation rate in the monthly thematic meetings on physical activity. Information on reasons for non-participation was enquired from those women who refused to participate.

Information on the feasibility of the counseling was collected by structured telephone interviews to the nurses of the trial clinics after the trial. The interview included questions on perceived pros and cons related to the contents and procedure of counseling and data collection as well as the nurse's willingness to utilize the approach in their work. The questions were tested in the pilot study and modified for this purpose.

Power calculations and sample size

The randomization of maternity clinics as clusters instead of women as individuals was taken into account in the power calculations [66]. When planning the RCT, no published studies on prevention of GDM among women at risk or data on decrease in the incidence of GDM were available. The power calculations for pair-matched study were based on the assumption of detecting a 40% reduction of incidence of GDM from 40% in the control clinics to 24% in the trial clinics. The power of the study was 0.80, significance level 0.05 and coefficient of variation of rate between clusters, indicating cluster sampling, 0.1. The dropout rate during the study (estimated 25%) was taken into account in the sample size calculations. Thus, a total number of 560 women should be recruited to the study. The number of women in the end of the study would thus be 210 + 210 (7+7 clinics, 30 women/clinic).

Success in recruitment and proportion of drop-outs (25%) were estimated based on the pilot study [28,67]. Our recruitment target seemed feasible since the annual number of deliveries in Pirkanmaa region was approximately 5000 [31] and at least fourth of these women were estimated to fulfill the inclusion criteria of this study.

The sample available for the 1-year follow-up (n = 462) is adequate to discover 5-20% difference of metabolic syndrome in trial and control groups. Intracluster correlation coefficient in the current study is estimated as 0.12 (valid when 0.10-0.30) [68].

Statistical analysis

The main method in the comparison of the trial and the control group is the multilevel analysis. In the multilevel analysis, both individual-level, nurse-level and clinic-level influences on the outcomes can be examined simultaneously and the results are corrected for between-clinic and between-nurse variation. Also non-parametric methods will be utilized when applicable. The choice of method depends on the measurement scale and distribution of the outcome variable. Intention-to-treat principle is followed, i.e. the analysis is performed in originally randomized groups. Information on drop-outs is received through linkage to birth registry. Intention-to-treat analyses are performed after birth registry linkage. A secondary analysis is performed without the drop-outs.

Discussion

The trial is expected to be effective in short-term prevention of gestational diabetes. The possible long-term benefits include prevention of chronic diseases, such as type 2 diabetes and cardiovascular diseases, both in mother and offspring.

The design of the study was a cluster-randomized trial instead of individual randomization. Contamination of nurses counseling practices would have been larger if individuals had been randomized instead of maternity clinic areas. Even though cluster randomization requires larger sample size, the results may be more easily applied to health care. Our purpose was not to build a design with maximal support for lifestyle modification, because it would not have been applicable in real health care setting. Embedding the trial into ongoing maternal care we aimed to increase the effectiveness rather than efficacy of the intervention.

Finland is one of the countries with nationally covering maternity center network funded by public taxation. If our trial was proved to be a cost-effective way to prevent gestational and perinatal complications it might be applicable and might have large public health impact even in those countries with different maternity care systems.

Abbreviations

GDM: gestational diabetes mellitus; BMI: body mass index; OGTT: oral glucose tolerance test; RCT: randomized controlled trial; AUC: area under curve; RPE: visual scale of perceived exertion; LTPA: leisure time physical activity; MET: multiples of resting metabolic equivalents; FFQ: food frequency questionnaire; QALY: quality adjusted life years

Acknowledgements

The main sources of funding in this study are Diabetes research fund, Competitive research funding from Pirkanmaa hospital district (EVO/TAYS), Academy of Finland, Ministry of Education, Ministry of Social Affairs and Health, all in Finland.
Professor Kaisa Kauppinen and MSc(sociology) Aino Luotonen have designed the questions concerning workability and return to work in the one-year follow-up questionnaire. Dr Mikael Fogelhom is acknowledged for his help in nutritional questions when planning the study. MSc Satu Lamberg participated in the beginning of the practical trial stage and planning of quality of life measurements. Ms Päivi Viitanen has participated to data collection and coding throughout the process. Professor Leena Hilakivi-Clarke has contributed to the planning of the blood samples and will perform blood sample analyses. The following persons have given important advice during the project: Dr Sirkku Tulokas (gestational diabetes), Dr Jukka Uotila (obstetrics), MSc Matti Pasanen (statistical questions and power calculations), Professor Hannu Oja (statistics), MSc Jani Raitanen (statistics), Dr Matti Salo (pediatrics), Dr Anna-Mari Aalto (quality of life), Dr Katinka Tuisku (depression scale), Dr Seppo Koskinen, Dr Tuija Martelin and Dr Päivikki Koponen (socioeconomic questions), Prof Suvi Virtanen (nutrition), Dr Tiina Solakivi (biochemistry). Mrs Ulla Hakala, Mrs Taru Helenius, Mrs Sirke Rasinperä and Ms Leena Aura from the UKK Institute laboratory have participated in the data collection.
Nurses from the following maternity centers are acknowledged for their partipation to the implementation of the trial: Pirkkala, Ylöjärvi, Akaa (Toijala, Viiala, Kylmäkoski), Kangasala, Hämeenkyrö, Mänttä-Vilppula, Nokia, Virrat, Mouhijärvi, Lempäälä, Vesilahti, Orivesi, Valkeakoski and Tampere (Muotiala).

Author details

[1]The UKK Institute for Health Promotion Research, Tampere, Finland. [2]National Institute for Health and Welfare, Helsinki, Finland. [3]Institute of Health and Society, Newcastle University, Newcastle upon Tyne, UK.

Authors' contributions

RL originated the idea to the study and has been project leader since pilot study. TK and MA are responsible for planning the counseling and preparing all material related to the counseling and training the nurses for counseling. MA designed the thematic meetings on physical activity and their material. KO designed the group exercise and related material to the meetings. MA and ET prepared the questions on participation of structured exercise for the 1-year follow-up questionnaire. KM is responsible for the physical measurements and laboratory tests in collaboration with TV, who is in charge of the lipid analyses. PK prepared the first version of the manuscript. All authors (RL, TK, MA, KO, KM, ET, PK and TV) have participated in drafting of the manuscript and approved the final manuscript.

Competing interests

We certify that none of us has affiliations with or involvement in any organization or entity with a direct financial interest in the subject matter of the study (e.g. employment, consultancies, stock ownership, honoraria, expert testimony).

Received: 29 January 2010 Accepted: 3 August 2010
Published: 3 August 2010

References

1. American Diabetes Association: **Gestational diabetes mellitus.** *Diabetes care* 2000, **23(Suppl 1)**:S77-79.
2. **Diagnosis and classification of diabetes mellitus.** *Diabetes care* 2006, **29(Suppl 1)**:S43-48.
3. Kaaja RJ, Poyhonen-Alho MK: **Insulin resistance and sympathetic overactivity in women.** *Journal of hypertension* 2006, **24(1)**:131-141.
4. Ben-Haroush A, Yogev Y, Hod M: **Epidemiology of gestational diabetes mellitus and its association with Type 2 diabetes.** *Diabet Med* 2004, **21(2)**:103-113.
5. STAKES The National Institute for Health and Welfare: *Finnish Medical Birth Registry* 2004.
6. O'Sullivan JB: **Diabetes mellitus after GDM.** *Diabetes* 1991, **40(Suppl 2)**:131-135.
7. Kaaja RJ, Greer IA: **Manifestations of chronic disease during pregnancy.** *Jama* 2005, **294(21)**:2751-2757.
8. Vohr BR, Boney CM: **Gestational diabetes: the forerunner for the development of maternal and childhood obesity and metabolic syndrome?** *J Matern Fetal Neonatal Med* 2008, **21(3)**:149-157.
9. Feig DS, Zinman B, Wang X, Hux JE: **Risk of development of diabetes mellitus after diagnosis of gestational diabetes.** *Cmaj* 2008, **179(3)**:229-234.
10. Lauenborg J, Mathiesen E, Hansen T, Glumer C, Jorgensen T, Borch-Johnsen K, Hornnes P, Pedersen O, Damm P: **The prevalence of the metabolic syndrome in a danish population of women with previous gestational diabetes mellitus is three-fold higher than in the general population.** *The Journal of clinical endocrinology and metabolism* 2005, **90(7)**:4004-4010.
11. Lawler DA, Fraser A, Lindsay RS, Ness A, Dabelea D, Catalano P, Davey Smith G, Sattar N, Nelson SM: **Association of existing diabetes, gestational diabetes and glycosuria in pregnancy with macrosomia and offspring body mass index, waist and fat mass in later childhood: findings from a prospective pregnancy cohort.** *Diabetologia* 2010, **53(1)**:89-97.
12. Clausen TD, Mathiesen ER, Hansen T, Pedersen O, Jensen DM, Lauenborg J, Schmidt L, Damm P: **Overweight and the metabolic syndrome in adult offspring of women with diet-treated gestational diabetes mellitus or type 1 diabetes.** *The Journal of clinical endocrinology and metabolism* 2009, **94(7)**:2464-2470.
13. Abrams B, Parker J: **Overweight and pregnancy complications.** *International journal of obesity* 1988, **12(4)**:293-303.
14. Moses RG, Shand JL, Tapsell LC: **The recurrence of gestational diabetes: could dietary differences in fat intake be an explanation?** *Diabetes care* 1997, **20(11)**:1647-1650.
15. Wang Y, Storlien LH, Jenkins AB, Tapsell LC, Jin Y, Pan JF, Shao YF, Calvert GD, Moses RG, Shi HL, *et al*: **Dietary variables and glucose tolerance in pregnancy.** *Diabetes care* 2000, **23(4)**:460-464.
16. Bo S, Menato G, Lezo A, Signorile A, Bardelli C, De Michieli F, Massobrio M, Pagano G: **Dietary fat and gestational hyperglycaemia.** *Diabetologia* 2001, **44(8)**:972-978.
17. Saldana TM, Siega-Riz AM, Adair LS: **Effect of macronutrient intake on the development of glucose intolerance during pregnancy.** *The American journal of clinical nutrition* 2004, **79(3)**:479-486.

18. Clapp JF: **Exercise during pregnancy. A clinical update.** *Clinics in sports medicine* 2000, **19(2)**:273-286.
19. Iqbal R, Rafique G, Badruddin S, Qureshi R, Cue R, Gray-Donald K: **Increased body fat percentage and physical activity are independent predictors of gestational diabetes mellitus in South Asian women.** *European journal of clinical nutrition* 2007, **61**:736-742.
20. Dempsey JC, Sorensen TK, Williams MA, Lee IM, Miller RS, Dashow EE, Luthy DA: **Prospective study of gestational diabetes mellitus risk in relation to maternal recreational physical activity before and during pregnancy.** *American journal of epidemiology* 2004, **159(7)**:663-670.
21. Oken E, Ning Y, Rifas-Shiman SL, Radesky JS, Rich-Edwards JW, Gillman MW: **Associations of physical activity and inactivity before and during pregnancy with glucose tolerance.** *Obstetrics and gynecology* 2006, **108(5)**:1200-1207.
22. Dempsey JC, Butler CL, Sorensen TK, Lee IM, Thompson ML, Miller RS, Frederick IO, Williams MA: **A case-control study of maternal recreational physical activity and risk of gestational diabetes mellitus.** *Diabetes research and clinical practice* 2004, **66(2)**:203-215.
23. **Impact of physical activity during pregnancy and postpartum on chronic disease risk.** *Medicine and science in sports and exercise* 2006, **38(5)**:989-1006.
24. Ratner RE: **Prevention of type 2 diabetes in women with previous gestational diabetes.** *Diabetes care* 2007, **30(Suppl 2)**:S242-245.
25. Mottola M, Lander S, Giroux I, Hammond J, Lebrun C, McManus R, Sopper M: **Glucose and insulin responses in women at risk for GDM before and after a nutrition, exercise & lifestyle intervention program (NELIP).** *Medicine and science in sports and exercise* 2005, **37(5)**:309-310.
26. Hui A, Ludwig S, Gardiner P, Sevenhuysen G, Murray R, Morris M, Shen G: **Community-based exercise and dietary intervention during pregnancy: a pilot study.** *Can J Diabetes* 2006, **30(2)**:169-175;130:169-175.
27. Kinnunen TI, Pasanen M, Aittasalo M, Fogelholm M, Hilakivi-Clarke L, Weiderpass E, Luoto R: **Preventing excessive weight gain during pregnancy - a controlled trial in primary health care.** *European journal of clinical nutrition* 2007, **61(7)**:884-891.
28. Aittasalo M, Pasanen M, Fogelholm M, Kinnunen TI, Ojala K, Luoto R: **Physical activity counseling in maternity and child health care - a controlled trial.** *BMC women's health* 2008, **8**:14.
29. Chasan-Taber L, Marcus BH, Stanek E, Ciccolo JT, Marquez DX, Solomon CG, Markenson G: **A randomized controlled trial of prenatal physical activity to prevent gestational diabetes: design and methods.** *Journal of women's health (2002)* 2009, **18(6)**:851-859.
30. Oostdam N, van Poppel MN, Eekhoff EM, Wouters MG, van Mechelen W: **Design of FitFor2 study: the effects of an exercise program on insulin sensitivity and plasma glucose levels in pregnant women at high risk for gestational diabetes.** *BMC pregnancy and childbirth* 2009, **9**:1.
31. Hakulinen-Viitanen T, Pelkonen M, A H: **Äitiys- ja lastenneuvolatyö Suomessa. Sosiaali- ja terveysministeriön selvityksiä 2005:22 (in Finnish).** Helsinki: Ministry of Social Affairs and Health 2005.
32. Viisainen K, (ed.): **Seulontatutkimukset ja yhteistyö äitiyshuollossa. Suositukset 1999. Stakes oppaita 34 (in Finnish).** Helsinki: Gummerus 1999.
33. Laitakari J, Asikainen TM: **How to promote physical activity through individual counseling–a proposal for a practical model of counseling on health-related physical activity.** *Patient Educ Couns* 1998, **33(1 Suppl)**: S13-24.
34. Green LW, Kreuter MW, Deeds SG, Partridge KB: **Health education planning. A diagnostic approach.** Palo Alto, CA: Mayfield 1980.
35. Prochaska JO, Velicer WF: **The transtheoretical model of health behavior change.** *Am J Health Promot* 1997, **12(1)**:38-48.
36. Aittasalo M, Miilunpalo S, Suni J: **The effectiveness of physical activity counseling in a work-site setting. A randomized, controlled trial.** *Patient Educ Couns* 2004, **55(2)**:193-202.
37. Institute of Medicine: **Nutrition during pregnancy, weight gain and nutrient supplements. Report of the Subcommittee on Nutritional Status and Weight Gain during Pregnancy, Subcommittee on Dietary Intake and Nutrient Supplements during Pregnancy, Committee on Nutritional Status during Pregnancy and Lactation, Food and Nutrition Board.** Washington, DC: National Academy Press 1990.
38. Althuizen E, van Poppel MN, Seidell JC, van der Wijden C, van Mechelen W: **Design of the New Life(style) study: a randomised controlled trial to optimise maternal weight development during pregnancy. [ISRCTN85313483].** *BMC public health* 2006, **6**:168.
39. **ACOG Committee opinion. Number 267, January 2002: exercise during pregnancy and the postpartum period.** *Obstetrics and gynecology* 2002, **99(1)**:171-173.
40. Artal R, O'Toole M: **Guidelines of the American College of Obstetricians and Gynecologists for exercise during pregnancy and the postpartum period.** *British journal of sports medicine* 2003, **37(1)**:6-12, discussion 12.
41. Davies GA, Wolfe LA, Mottola MF, MacKinnon C, Arsenault MY, Bartellas E, Cargill Y, Gleason T, Iglesias S, Klein M, *et al*: **Exercise in pregnancy and the postpartum period.** *J Obstet Gynaecol Can* 2003, **25(6)**:516-529.
42. Pate RR, Pratt M, Blair SN, Haskell WL, Macera CA, Bouchard C, Buchner D, Ettinger W, Heath GW, King AC, *et al*: **Physical activity and public health. A recommendation from the Centers for Disease Control and Prevention and the American College of Sports Medicine.** *Jama* 1995, **273(5)**:402-407.
43. **Physical Activity Guidelines Advisory Committee report, 2008.** *Nutr Rev* 2009, **67(2)**:114-20.
44. American College of Sports Medicine Position Stand: **The recommended quantity and quality of exercise for developing and maintaining cardiorespiratory and muscular fitness, and flexibility in healthy adults.** *Medicine and science in sports and exercise* 1998, **30(6)**:975-991.
45. Borg G: **Perceived exertion as an indicator of somatic stress.** *Scandinavian journal of rehabilitation medicine* 1970, **2(2)**:92-98.
46. Borg GA: **Psychophysical bases of perceived exertion.** *Medicine and science in sports and exercise* 1982, **14(5)**:377-381.
47. Haskell WL, Lee IM, Pate RR, Powell KE, Blair SN, Franklin BA, Macera CA, Heath GW, Thompson PD, Bauman A: **Physical activity and public health: updated recommendation for adults from the American College of Sports Medicine and the American Heart Association.** *Circulation* 2007, **116(9)**:1081-1093.
48. Howley ET: **Type of activity: resistance, aerobic and leisure versus occupational physical activity.** *Medicine and science in sports and exercise* 2001, **33(6 Suppl)**:S364-369, discussion S419-320.
49. **Nordic Nutrition Recommendations. Integrating nutrition and physical activity.** Copenhagen: Nordic Council of Ministers 2004.
50. Valtion ravitsemusneuvottelukunta: **Suomalaiset ravitsemussuositukset 2005 - ravinto ja liikunta tasapainoon (in Finnish).** Helsinki: Edita 2005.
51. Tuomilehto J, Lindstrom J, Eriksson JG, Valle TT, Hamalainen H, Ilanne-Parikka P, Keinanen-Kiukaanniemi S, Laakso M, Louheranta A, Rastas M, *et al*: **Prevention of type 2 diabetes mellitus by changes in lifestyle among subjects with impaired glucose tolerance.** *The New England journal of medicine* 2001, **344(18)**:1343-1350.
52. **National Cholesterol Education Program Expert Panel on Detection, Evaluation, and Treatment of High blood cholesterol in adults.** *JAMA* 2001, **285**:2486-2497.
53. Tudor-Locke C, Henderson KA, Wilcox S, Cooper RS, Durstine JL, Ainsworth BE: **In their own voices: definitions and interpretations of physical activity.** *Womens Health Issues* 2003, **13(5)**:194-199.
54. Aittasalo M, Pasanen M, Fogelholm M, Ojala K: **Validity and repeatability of a short pregnancy leisure time physical activity questionnaire.** *J Phys Act Health* 2010, **7(1)**:109-18.
55. Erkkola M, Karppinen M, Javanainen J, Rasanen L, Knip M, Virtanen SM: **Validity and reproducibility of a food frequency questionnaire for pregnant Finnish women.** *American journal of epidemiology* 2001, **154(5)**:466-476.
56. Sintonen H: **The 15 D instrument of health-related quality of life: properties and applications.** *Annals of medicine* 2001, **33(5)**:328-336.
57. Benjamins MR, Hummer RA, Eberstein IW, Nam CB: **Self-reported health and adult mortality risk: an analysis of cause-specific mortality.** *Social science & medicine (1982)* 2004, **59(6)**:1297-1306.
58. Aalto A-M, Aro S, Aro A, Mähönen M: **"RAND 36-item health survey 1.0". Suomenkielinen versio terveyteen liittyvän elämänlaadun kyselystä. STAKESin Aiheita-sarja 2/1995 (in Finnish).** Helsinki: STAKES 1995.
59. Beck A, Steer R, Brown G: **Manual for the Beck Depression Inventory-II.** San Antonio: TX: Psychological Corporation 1996.
60. Kujala V, Tammelin T, Remes J, Vammavaara E, Ek E, Laitinen J: **Work ability index of young employees and their sickness absence during the following year.** *Scandinavian journal of work, environment & health* 2006, **32(1)**:75-84.
61. Kirwan JP, Huston-Presley L, Kalhan SC, Catalano PM: **Clinically useful estimates of insulin sensitivity during pregnancy: validation studies in**

women with normal glucose tolerance and gestational diabetes **mellitus.** *Diabetes care* 2001, **24(9)**:1602-1607.
62. Teramo K, Leinonen P: **Diabetes ja raskaus.** *Naistentaudit ja synnytykset* Helsinki: DuodecimYlikorkala O, Kauppila A 1996, 513-522.
63. Duodecim : **Finnish guidelines for gestational diabetes.** 2008, **124**:1556-1569[http://www.terveysportti.fi/xmedia/extra/hoi/hoi50068.pdf].
64. Le Floch JP, Escuyer P, Baudin E, Baudon D, Perlemuter L: **Blood glucose area under the curve. Methodological aspects.** *Diabetes care* 1990, **13(2)**:172-175.
65. Drummond MF, Sculpher MJ, Torrance GW, O'Brien BJ, Stoddart GL: **Methods for the economic evaluation of health care programmes.** New York: Oxford University press, Third 2005.
66. Campbell MK, Mollison J, Steen N, Grimshaw JM, Eccles M: **Analysis of cluster randomized trials in primary care: a practical approach.** *Family practice* 2000, **17(2)**:192-196.
67. Kinnunen TI, Aittasalo M, Koponen P, Ojala K, Mansikkamaki K, Weiderpass E, Fogelholm M, Luoto R: **Feasibility of a controlled trial aiming to prevent excessive pregnancy-related weight gain in primary health care.** *BMC pregnancy and childbirth* 2008, **8**:37.
68. Campbell MK, Thomson S, Ramsay CR, MacLennan GS, Grimshaw JM: **Sample size calculator for cluster randomized trials.** *Computers in biology and medicine* 2004, **34(2)**:113-125.

Pre-publication history

The pre-publication history for this paper can be accessed here:
http://www.biomedcentral.com/1471-2393/10/39/prepub

doi:10.1186/1471-2393-10-39
Cite this article as: Luoto *et al.*: Prevention of Gestational Diabetes: Design of a Cluster-Randomized Controlled Trial and One-Year Follow-Up. *BMC Pregnancy and Childbirth* 2010 **10**:39.

Submit your next manuscript to BioMed Central and take full advantage of:

- **Convenient online submission**
- **Thorough peer review**
- **No space constraints or color figure charges**
- **Immediate publication on acceptance**
- **Inclusion in PubMed, CAS, Scopus and Google Scholar**
- **Research which is freely available for redistribution**

Submit your manuscript at
www.biomedcentral.com/submit

BioMed Central
