# Supplementary material for: Primary Prevention of Gestational Diabetes Mellitus and Large-for-Gestational-Age Newborns by Lifestyle Counseling: A Cluster-Randomized Controlled Trial
Source: PLoS Med. 2011 May 17;8(5):e1001036. doi: 10.1371/journal.pmed.1001036 (PMC3096610; doi:10.1371/journal.pmed.1001036)
Supplement: Text S2 — CONSORT checklist. (DOC) [file pmed.1001036.s007.doc]

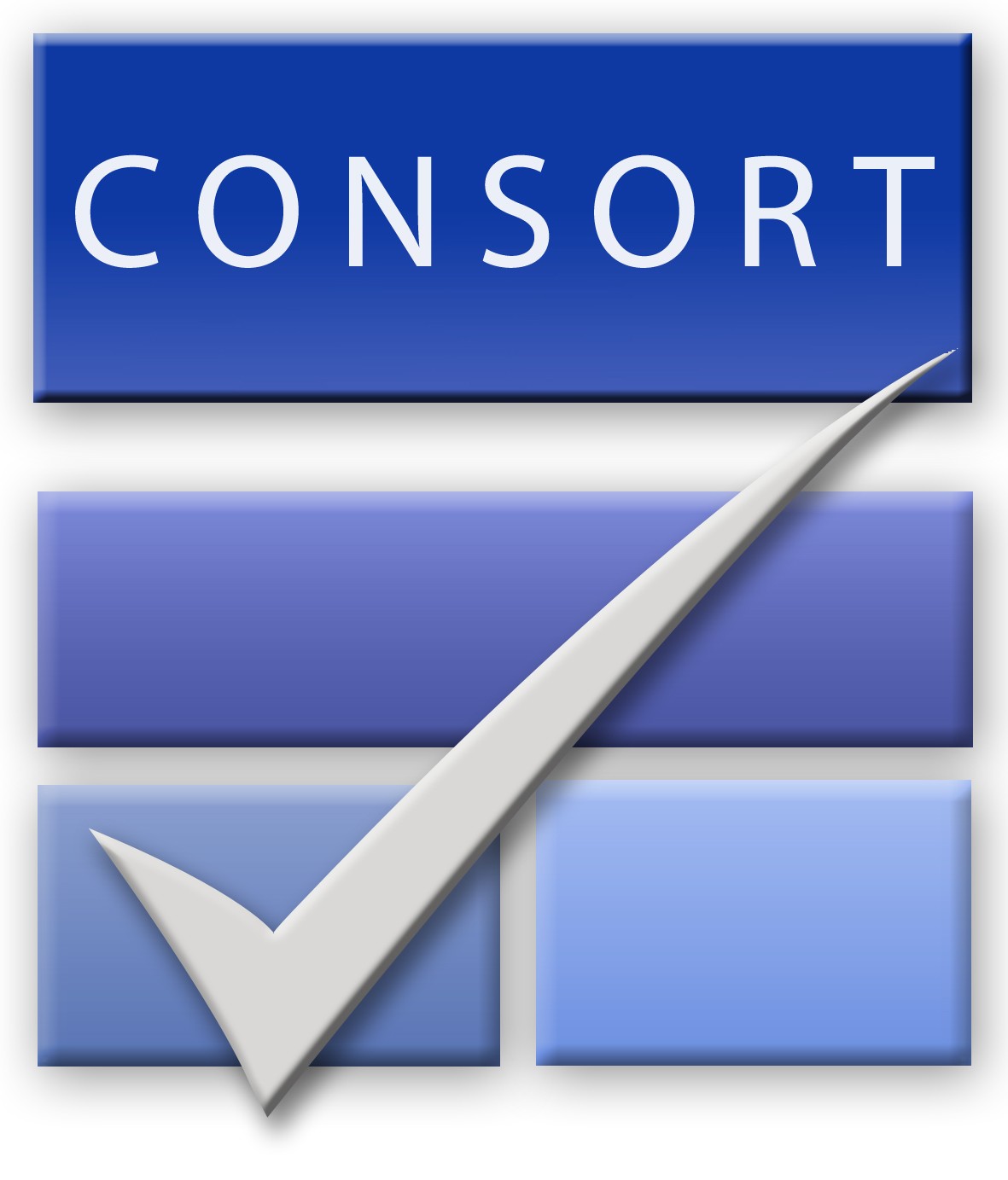
CONSORT 2010 checklist of information to include when reporting a randomised trial*

(extended with checklists for non-pharmacologic trials and cluster-randomized trials)

| Section/Topic | Item No | Checklist item | Reported on page No |
| --- | --- | --- | --- |
| Title and abstract | | | |
|  | 1a | Identification as a randomised trial in the title | 1,2 |
|  | 1b | Structured summary of trial design, methods, results, and conclusions (for specific guidance see CONSORT for abstracts) | 2 |
|  | **Extension for Non pharmacologic Trials**: In the abstract, description of the experimental treatment, comparator, care providers, centers and blinding status  **Extension for Cluster randomized trials**: *specifying that allocation was based on clusters* | | 2 |
| Introduction | | | |
| Background and objectives | 2a | Scientific background and explanation of rationale *(including the rationale for using a cluster design)* | 3-4 |
|  | 2b | Specific objectives or hypotheses | 3 |
| Methods | | | |
| Trial design | 3a | Description of trial design (such as parallel, factorial) including allocation ratio | 4 |
|  | 3b | Important changes to methods after trial commencement (such as eligibility criteria), with reasons | - |
| Participants | 4a | Eligibility criteria for participants *and clusters* | 4 |
|  | 4b | Settings and locations where the data were collected | 4 |
|  | **Extension for Non pharmacologic Trials**: When applicable, eligibility criteria for centers and those performing the interventions | | 4 |
| Interventions | 5 | The interventions for each group with sufficient details to allow replication, including how and when they were actually administered; *whether they pertain to the individual level, the cluster level, or both* | 4 |
|  | **Extension for Non pharmacologic Trials**: Precise details of both the experimental treatment and comparator | | 4-5 |
|  | **Extension for Non pharmacologic Trials**: Description of the different components of the interventions and when applicable, descriptions of the procedure for tailoring the interventions to individual participants | | 4-5 |
|  | **Extension for Non pharmacologic Trials**: Details of how the interventions were standardized | | 4-5 |
|  | **Extension for Non pharmacologic Trials**: Details of how adherence of care providers with the protocol was assessed or enhanced | | 4, 6,8, Table 4 |
| Outcomes | 6a | Completely defined pre-specified primary and secondary outcome measures, *whether they pertain to the individual level, the cluster level, or both*, including how and when they were assessed | 5-6 |
|  | 6b | Any changes to trial outcomes after the trial commenced, with reasons | - |
| Sample size | 7a | How sample size was determined; *method of calculation, number of clusters, cluster size, a coefficient of intracluster correlation (ICC or k) and an indication of its uncertainty)* | 6 |
|  | 7b | When applicable, explanation of any interim analyses and stopping guidelines | - |
|  | **Extension for Non pharmacologic Trials**: When applicable, details of whether and how the clustering by care providers or centers was addressed | | 3-4 |
| Randomisation: |  |  |  |
| Sequence generation | 8a | Method used to generate the random allocation sequence | 4 |
|  | 8b | Type of randomisation; details of any restriction (such as blocking and block size) | 4 |
|  | **Extension for Non pharmacologic Trials**: When applicable, how care providers were allocated to each trial group | | 4 |
| Allocation concealment mechanism | 9 | Mechanism used to implement the random allocation sequence (such as sequentially numbered containers), describing any steps taken to conceal the sequence until interventions were assigned; *specifying that allocation was based on clusters rather than individuals* | 4 |
| Implementation | 10 | Who generated the random allocation sequence, who enrolled participants, and who assigned participants to interventions | 4 |
| Blinding | 11a | If done, who was blinded after assignment to interventions (for example, participants, care providers, those assessing outcomes) and how | - |
|  | 11b | If relevant, description of the similarity of interventions | - |
|  | **Extension for Non pharmacologic Trials:** Whether or not those administering co-interventions were blinded to group assignment | | -- |
|  | **Extension for Non pharmacologic Trials:** If blinded method of blinding and description of the similarity of interventions | | - |
| Statistical methods | 12a | Statistical methods used to compare groups for primary and secondary outcomes; *indicating how clustering was taken into account* | 6-7 |
|  | 12b | Methods for additional analyses, such as subgroup analyses and adjusted analyses | 6-7 |
|  | **Extension for Non pharmacologic Trials:** When applicable, details of whether and how the clustering by care providers or centers was addressed | | 4 |
| Results | | | |
| Participant flow (a diagram is strongly recommended) | 13a | For each group, the numbers of participants who were randomly assigned, received intended treatment, and were analysed for the primary outcome; *flow of clusters* | 7, Figure 1 |
|  | 13b | For each group *(cluster)*, losses and exclusions after randomisation, together with reasons | 7, Figure 1 |
|  | **Extension for Non pharmacologic Trials:** The number of care providers or centers performing the intervention in each group and the number of patients treated by each care provider or in each center | | 7, Figure 1 |
| Implementation of intervention | **Extension for Non pharmacologic Trials:** Details of the experimental treatment and comparator as they were implemented | | 4-5 |
| Recruitment | 14a | Dates defining the periods of recruitment and follow-up | 4 |
|  | 14b | Why the trial ended or was stopped | - |
| Baseline data | 15 | A table showing baseline demographic and clinical characteristics for each group; *for the individual and cluster level as applicable* | Table 1,  Table S1 |
|  | **Extension for Non pharmacologic Trials:** When applicable, a description of care providers (case volume, qualification, expertise etc.) and centers (volume) in each group | | 4 |
| Numbers analysed | 16 | For each group, number of *clusters and* participants (denominator) included in each analysis and whether the analysis was by original assigned groups | 6-7 |
| Outcomes and estimation | 17a | For each primary and secondary outcome, results for each group, *for the individual or cluster level as applicable*, and the estimated effect size and its precision (such as 95% confidence interval) *and a coefficient of intracluster correlation (ICC or k) for each primary outcome* | 8 |
|  | 17b | For binary outcomes, presentation of both absolute and relative effect sizes is recommended | Table 2,  Table 3 |
| Ancillary analyses | 18 | Results of any other analyses performed, including subgroup analyses and adjusted analyses, distinguishing pre-specified from exploratory | 8-9 |
| Harms | 19 | All important harms or unintended effects in each group (for specific guidance see CONSORT for harms) | 10, Table 5 |
| Discussion | | | |
| Limitations | 20 | Trial limitations, addressing sources of potential bias, imprecision, and, if relevant, multiplicity of analyses | 10-11 |
| Generalizability | 21 | Generalizability (external validity, applicability) *to individuals and/or clusters*, of the trial findings | 11 |
|  | **Extension for Non pharmacologic Trials:** Generalizability (external validity) of the trial findings according to the intervention, comparators, patients, and care providers and centers involved in the trial | | 11 |
| Interpretation | 22 | Interpretation consistent with results, balancing benefits and harms, and considering other relevant evidence | 11 |
|  | **Extension for Non pharmacologic Trials:** In addition, take into account the choice of the comparator, lack of or partial blinding, and unequal expertise of care providers or centers in each group | | 11 |
| Other information | | |  |
| Registration | 23 | Registration number and name of trial registry | 2 |
| Protocol | 24 | Where the full trial protocol can be accessed, if available | TextS2 |
| Funding | 25 | Sources of funding and other support (such as supply of drugs), role of funders | 18 |

*We strongly recommend reading this statement in conjunction with the CONSORT 2010 Explanation and Elaboration for important clarifications on all the items. If relevant, we also recommend reading CONSORT extensions for cluster randomised trials, non-inferiority and equivalence trials, non-pharmacological treatments, herbal interventions, and pragmatic trials. Additional extensions are forthcoming: for those and for up to date references relevant to this checklist, see [www.consort-statement.org](http://www.consort-statement.org/)
